# Coagulation cascade and complement system in systemic lupus erythematosus

**DOI:** 10.18632/oncotarget.23206

**Published:** 2017-12-11

**Authors:** Yan Liang, Shang-Bo Xie, Chang-Hao Wu, Yuan Hu, Qin Zhang, Si Li, Yin-Guang Fan, Rui-Xue Leng, Hai-Feng Pan, Hua-Bao Xiong, Dong-Qing Ye

**Affiliations:** ^1^ Department of Epidemiology and Biostatistics, School of Public Health, Anhui Medical University, Hefei, Anhui, PR China; ^2^ BGI-Shenzhen, Shenzhen, China; ^3^ Department of Biochemical Sciences, Faculty of Health and Medical Sciences, University of Surrey, Guildford, Surrey, GU2 7XH, UK; ^4^ Department of Medicine, Immunology Institute, Icahn School of Medicine at Mount Sinai, New York, NY, USA; ^5^ Department of Epidemiology and Biostatistics, School of Public Health, Anhui Medical University, Hefei, Anhui, PR China; ^6^ Anhui Province Key Laboratory of Major Autoimmune Diseases, Anhui, PR China

**Keywords:** systemic lupus erythematosus, coagulation cascade, complement system, biomarker, omics

## Abstract

This study was conducted to (1) characterize coagulation cascade and complement system in systemic lupus erythematosus (SLE); (2) evaluate the associations between coagulation cascade, complement system, inflammatory response and SLE disease severity; (3) test the diagnostic value of a combination of D-dimer and C4 for lupus activity. Transcriptomics, proteomics and metabolomics were performed in 24 SLE patients and 24 healthy controls. The levels of ten coagulations, seven complements and three cytokines were measured in 112 SLE patients. Clinical data were collected from 2025 SLE patients. The analysis of multi-omics data revealed the common links for the components of coagulation cascade and complement system. The results of ELISA showed coagulation cascade and complement system had an interaction effect on SLE disease severity, this effect was pronounced among patients with excess inflammation. The analysis of clinical data revealed a combination of D-dimer and C4 provided good diagnostic performance for lupus activity. This study suggested that coagulation cascade and complement system become ‘partners in crime’, contributing to SLE disease severity and identified the diagnostic value of D-dimer combined with C4for lupus activity.

## INTRODUCTION

Systemic lupus erythematosus (SLE) is a systemic autoimmune disease that is characterized by a diverse array of autoantibody production, immune complex deposition and tissue and organ damage [1]. Previous studies have reported the dysregulation of coagulation and complement-related genes and proteins in patients with SLE, suggesting that coagulation cascade and complement system have a role in pathogenic process of SLE [2, 3]. However, most of these studies focused on individual molecules which limit sufficient insight into the complex disease, such as SLE. Technological advances in the expression profiling, such as transcriptomics, proteomics and metabolomics, have broadened the spectrum of detectable compounds [4]. These technologies are now beginning to be utilized in the study of SLE. Several studies have revealed the important roles of some signaling pathways in pathogenic process of SLE, including coagulation cascade and complement system [5–9]. These studies provide more information on the pathogenic pathways, but they are all based on a single omics platform and cannot reveal the links for the components of signaling pathways at transcript, protein and metabolite levels. Given that many signaling pathways are orchestrated by global networks that cut across multiple omics layers [10], the researchers have recently begun to integrate the multi-omics data measured in a certain experiment to give a much more detailed view of signaling pathways than when applied individually [11–13]. However, to date no researcher has comprehensively integrated these diverse sets of data to study SLE.

As fundamental biological processes, the individual constituents of coagulation cascade and complement system are finely orchestrated to form two distinct multi-component networks [14]. However, a study discovered a novel complement cleavage mechanism in an acute lung inflammatory injury model by which thrombin can efficiently cleave complement 5 (C5) in the absence of C3 [15]. This observation was soon extended to the multi-intercommunication between coagulation cascade and complement system by *in vivo* and *in vitro* experiments [16, 17]. Further, recent studies suggest that the interaction is not confined to the coagulation cascade and complement system. Specifically, the coagulation cascade and complement system can interact with each other indirectly through the regulation of inflammatory mediators [18–20]. For example, an interaction between C5a and inflammatory cytokines have been demonstrated, including an effect on the production of tumor necrosis factor (TNF)-α and interleukin (IL)-6 [21]. Then, these cytokines can enhance the activation of coagulation cascade by promoting the expression of coagulants and inhibiting the production of anticoagulants [22]. The interplays between complement system, coagulation cascade and inflammatory response have been reported to be associated with disease severity in various clinical and experimental settings [18, 19]. However, the potential role of these interactions for disease severity in the patients with SLE has yet to be determined.

Evidence shows that early detection combined with a timely use of disease modifying anti-rheumatic drugs (DMARDs) can improve the outcome of SLE. The challenge is therefore to identify the best biomarkers to detect and diagnose lupus activity. In clinical practice, SLE disease activity index (SLEDAI) is used to diagnose lupus activity [23]. SLEDAI has an extremely high sensitivity and specificity; however, one of the main limitations of SLEDAI was overly complex because measurements of SLEDAI often use blood biochemical tests combined with cerebrospinal fluid cytology studies, X-rays and assays of renal tissue markers [23]. Other limitations include the expensive cost and time-consuming process. Many recent publications have documented specific gene and/or protein as potential biomarker of lupus activity, including haptoglobin, alpha-1 anti-chymotrypsin, retinol binding protein, miR-21, miR-181a, miR-196a, human T cell immunoglobulin domain and mucin-3 (TIM-3), TIM-3 ligands, TNF-like weak inducer of apoptosis, CD72, programmed death ligand 1, ferritin, insulin-like growth factor binding protein 2, tumor necrosis factor receptor type II, homocysteine and B-lymphocyte stimulator [24–38]. An ideal lupus activity biomarker should be biologically relevant, reproducible, simple to apply in routine practice (inexpensive, easy and rapid to quantify, non-invasive) and would have a high degree of sensitivity and specificity. However, at present, no biomarker exists that fulfils all of the above. There is thus an urgent need for discovering new and reliable biomarkers. As most of the components of the coagulation cascade and complement system are present in peripheral blood and are readily accessible, they may be used as biomarker.

**Figure 1:**
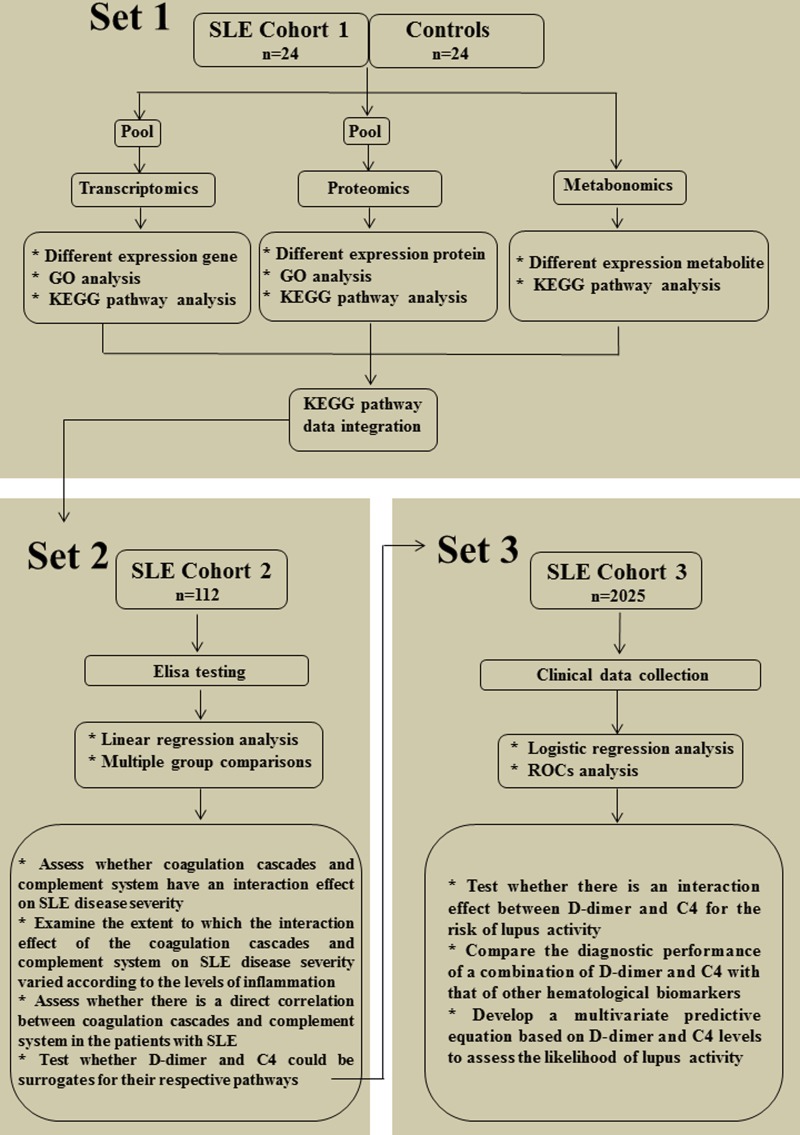
Scheme depicting the strategy used in this study Part 1 (multi-omics analysis): Transcriptomics, proteomics and metabolomics were performed simultaneouslyin SLE cohort I (*n* = 24) and healthy controls (*n* = 24) to systematically characterize coagulation cascade and complement system at transcript, protein and metabolite levels; Part 2 (protein expression analysis): The levels of coagulations, complements and cytokines were measured in SLE cohort II (*n* = 112) by using ELISA to evaluate the associations between coagulation cascade, complement system, inflammatory response and SLE disease severity; Part 3 (biomarker identification analysis): Clinical data from SLE cohort III (*n* = 2025) were collected by medical record review to evaluate the diagnostic value of a combination of D-dimer and C4 for lupus activity. SLE, systemic lupus erythematosus; GO, gene ontology; KEGG, kyoto encyclopedia of genes and genomes; ROCs, receiver operating characteristic curves.

This study aimed firstly to systematically characterize coagulation cascade and complement system at transcript, protein and metabolite levels in the lupus cohort I by multi-omics analysis. The second objective was to evaluate the associations between complement system, coagulation cascade, inflammatory response and SLE disease severity in the lupus cohort II by protein expression analysis. The final objective was to determine the diagnostic value of a combined D-dimer and C4 for lupus activity in the lupus cohort III by biomarker identification analysis. The diagrams of experimental design and workflow of the three-part study are depicted in Figure 1.

## RESULTS

### Subjects

Patients were recruited in 3 sets: the cohort I including 24 SLE patients for the multi-omics analyses, the cohort II including 112 SLE patients for the protein expression analysis and the cohort III including 2025 SLE patients for the biomarker identification analysis. Twenty-four sex- and age-matched healthy controls (HCs) with no history of SLE, other inflammatory/autoimmune disease or cancer were used as controls for multi-omics analyses. Clinical and laboratory characteristics of all study population are summarized in Table 1.

**Table 1:** Baseline characteristics of the study population

	SLE cohort 1 (*n =* 24)	SLE cohort 2 (*n =* 112)	SLE cohort 3 (*n =* 2025)	HC (*n =* 24)
Characteristics				
Age, median (range), years	30.5 (16–50)	39 (14–86)	37 (10–78)	30 (20–55)
Sex, female/male, *n*	24/0	107/5	1858/167	24/0
SLEDAI score, median (range)	11 (4–28)	10 (1–46)	10 (0–48)	
Clinical manifestations				
Mucocutaneous manifestations, *n* (%)	12 (50)	51 (46)	887 (44)	
Arthritis, *n* (%)	5 (21)	25 (22)	284 (13)	
Nephritis, *n* (%)	13 (54)	64 (57)	913 (45)	
Serositis, *n* (%)	6 (25)	20 (18)	402 (20)	
Vasculitis, *n* (%)	2 (8)	9 (8)	210 (10)	
Neuropsychiatric manifestations, *n* (%)	2 (8)	6 (5)	185 (9)	
Myositis, *n* (%)	1 (4)	0 (0)	50 (2)	
Laboratory measurements				
Anti-Sm, *n* (%)	8 (33)	31 (28)	619 (31)	
Anti-SSA/Ro, *n* (%)	17 (71)	68 (61)	1200 (59)	
Anti-SSB/La, *n* (%)	7 (29)	13 (12)	277 (14)	
Anti-RNP, *n* (%)	10 (42)	40 (36)	621 (31)	
Anti-Rib P, *n* (%)	8 (33)	29 (26)	436 (22)	
Anti-dsDNA, *n* (%)	10 (42)	51 (46)	781 (39)	
Thrombocytopenia, *n* (%)	11 (45)	21 (19)	499 (25)	
Leukopenia, *n* (%)	10 (42)	31 (28)	620 (31)	
Low complement C3, *n* (%)	22 (92)	80 (71)	1380 (68)	
Low complement C4, *n* (%)	16 (67)	52 (46)	999 (49)	
High ESR, *n* (%)	21 (88)	81 (72)	1426 (70)	
High CRP, *n* (%)	13 (54)	48 (43)	1111 (55)	
Immunosuppressive drugs, *n* (%)	9 (38)	61 (54)	1300 (64)	

SLE, systemic lupus erythematosus; HC, healthy subjects; SLEDAI, SLE Disease Activity Index; ESR, erythrocyte sedimentation rate; CRP, C reactive protein.

### Transcriptomics

An average of 22.3 million clean reads was generated per pool, of which 56.03%–63.39% could be uniquely aligned with the human genome 19 (hg19). Among the quantified genes, 420 genes showed significant change in expression pattern with 190 being up-regulated and 230 down-regulated in SLE patients when compared with HC (Supplementary Table 1). Enrichment analysis of these differentially expressed genes revealed gene ontology (GO) biological processes such as lymphocyte activation, defense response and leukocyte differentiation (Supplementary Table 2) as well as kyoto encyclopedia of genes and genomes (KEGG) pathways such as T cell receptor signaling, NF-kappa B signaling and, of particular note, coagulation cascade and complement system (Supplementary Table 3).

### Proteomics

**Figure 2:**
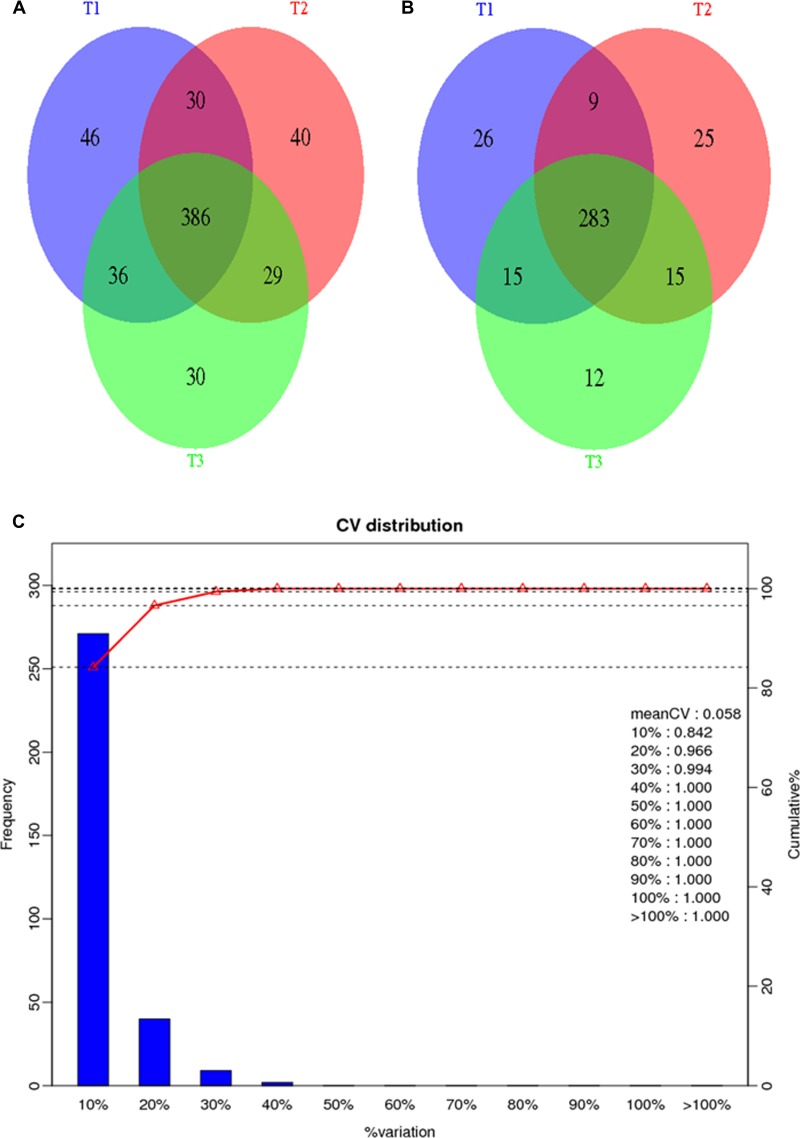
General information of the proteomics analysis (**A**) A Venn diagram representing the overlap of identified proteins among the three technical replicates; (**B**) A Venn diagram representing the overlap of quantified proteins among the three technical replicates; (**C**) Repeatability of quantification data among biological replicates.

For each group, three biological replicate samples of patient (SLE_1; SLE_2; SLE_3) and control (HC_1; HC_2; HC_3) were included in the isobaric tag for relative and absolute quantitation (iTRAQ) experiment. Additionally, three technical repeats (T1; T2; T3) were processed for each biological replicate. For the six samples, a total of 301389 (T1), 300097 (T2), 298878 (T3) mass spectra were generated. After data filtering, 21471 (T1), 20811 (T2), 20763 (T3) unique spectra that matched to special peptides were obtained. Through searching Mascot, a total of 2715 (T1), 2643 (T2), 2652 (T3) peptides, 2355 (T1), 2284 (T2), 2291 (T3) unique peptides and 498 (T1), 485 (T2), 481 (T3) proteins were identified. We combined the three technical replicates to identify 597 proteins (Figure 2A). Among all of the identified proteins, 385 proteins could be quantified (Figure 2B). The reproducibility of the iTRAQ experiment was evaluated by comparing the differences of quantification data among biological replicates. The results revealed more than 98% of the proteins exhibited differences of less than 20% (Figure 2C), suggesting the reproducibility was satisfactory. Among the quantified proteins, 87 proteins had increased expression and 24 proteins had decreased expression in SLE patients (Supplementary Table 4). GO enrichment analysis indicated the participation of differentially expressed proteins in a diverse array of biological function, including inflammatory response, sensory perception of light stimulus and cellular response to reactive oxygen species (Supplementary Table 5). KEGG pathway enrichment analysis identified coagulation cascade and complement system as the most relevant pathways (Supplementary Table 6).

### Metabolomics

A total of 195 significantly altered metabolites were found in SLE patients. Representative metabolites can be found as Supplementary Table 7. Through searching KEGG, the metabolic pathways that were altered in SLE patients have been identified, which involve steroid hormone biosynthesis, tryptophan metabolism, and, of interest, coagulation cascade and complement system (Supplementary Table 8).

### Integrative analysis of multi-omics data

**Figure 3:**
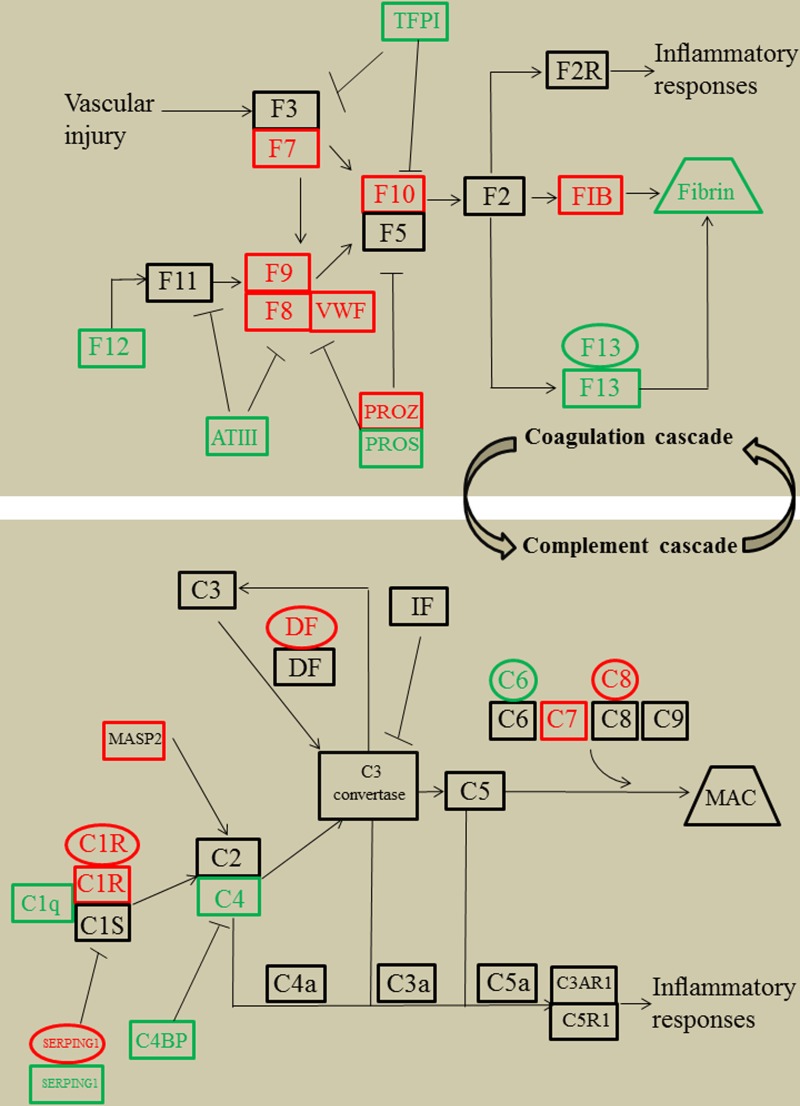
Simplified coagulation cascade and complement system featuring transcriptomics, proteomics, and metabolomics regulations of systemic lupus erythematosus patients compared with healthy controls, according to KEGG nomenclature Genes are presented as ovals, proteins as rectangles, metabolites by trapezoid. Regulation is color coded in which red stands for up regulated, green for down regulated, and black for unregulated. TFPI, tissue factor pathway inhibitor; F3, coagulation factor 3; F7, coagulation factor 7; F8, coagulation factor 8; F9, coagulation factor 9; F11, coagulation factor 11; F12, coagulation factor 12; F13, coagulation factor 13; F2, coagulation factor 2; F2R, F2 receptor; VWF, Von Willebrand factor; ATIII, antithrombin-III; PROZ, protein Z; PROS, protein S; FIB, fibrinogen; IF, complement factor I; DF, complement factor D; MAC, membrane attack complex; MASP2, mannan-binding lectin-associated serine protease 2; C4BP, C4b-binding protein; KEGG, kyoto encyclopedia of genes and genomes.

We performed a combined mapping of transcriptomic, proteomic and metabolic data to coagulation cascade and complement system. This analysis revealed the common links for the components of coagulation cascade and complement system at transcript, protein and metabolite levels (Figure 3). To explore functional roles of these pathways for SLE disease severity, several differentially expressed signaling proteins that were identified by our proteomic experiment were measured in lupus cohort II, including mannan-binding lectin-associated serine protease 2 (MASP2), C7, C1q, C4, coagulation factor 7 (F7), F9, F12, F13, fibrinogen (FIB), Von Willebrand factor (VWF), protein S (PPOS) and antithrombin-III (ATIII). To compensate for the limitation of detection range of the proteomic technology, we also measured C3a, C4a, C5a, factor I (FI), thrombin-antithrombin complex (TAT) and D-dimer, all of which were important components of the coagulation cascade and complement system [2, 3]. Given that the interplays between the coagulation cascade, complement system and inflammatory response have been widely described [18, 19], three inflammatory cytokines were measured, including TNF-RII, IL-6 and IL-8. We then examined the associations between coagulation cascade, complement system, inflammatory response and SLE disease severity.

### The interaction effect between coagulation cascade and complement system for SLE disease severity

We performed 3 different analyses to assess the principal effects of coagulation cascade and complement system and their interaction effect on SLE disease severity in lupus cohort II. First, both the coagulation score (β = 0.706, 95% CI 0.371 to 1.040, *P* < 0.001) and complement score (β = 0.590, 95% CI 0.328 to 0.852, *P* < 0.001) were significantly associated with log-transformed (lt)SLEDAI when both were included in a linear regression model.

Second, a formal interaction test between coagulation score and complement score for ltSLEDAI yielded a statistically significant result (*P* < 0.001).

Finally, to help understand the interaction effect, we dichotomized coagulation score and complement score into two groups, respectively. This analysis yielded four groups for stratification of ltSLEDAI as described under ‘materials and methods’. The interaction effect is illustrated in Table 2. Specifically, in the group of low complement score, the relationship between coagulation score and ltSLEDAI was weak; the difference in mean value of ltSLEDAI between the low with the high coagulation score group was only 0.334. This difference increased to 0.831 in the group of high complement score. Similarly, in the group of low coagulation score, the relationship between complement score and ltSLEDAI was weak; the difference in mean value of ltSLEDAI between the low with the high complement score group was only 0.436. This difference increased to 0.933 in the group of high coagulation score. These results demonstrate that there is an interaction effect between coagulation cascade and complement system for SLE disease severity.

**Table 2:** Comparison of ltSLEDAI in groups of patients by high/low levels of coagulation score and complement score

	High coagulation score	Low coagulation score	Row *P* value (across coagulation score)
High complement score			
Patients, *n*	31	25	
Complement score, mean ± SD	0.892 ± 0.270	0.947 ± 0.367	
Coagulation score, mean ± SD	0.797 ± 0.263	0.117 ± 0.212	
ltSLEDAI, mean ± SD	2.894 ± 0.539	2.063 ± 0.725	0.001^a^
Low complement score			
Patients, *n*	25	31	
Complement score, mean ± SD	0.102 ± 0.200	-0.041 ± 0.301	
Coagulation score, mean ± SD	0.786 ± 0.318	0.116 ± 0.194	
ltSLEDAI, mean ± SD	1.961 ± 0.664	1.627 ± 0.899	0.523^a^
Column *P* value (across complement score)			
ltSLEDAI	0.001^a^	0.263^a^	0.001^a^

^a^Changes in ltSLEDAI are statistically compared using Tamhane’s T2(M) test. SLE, systemic lupus erythematosus; SLEDAI, SLE Disease Activity Index; ltSLEDAI, log-transformed SLEDAI.

### The interaction effect between the coagulation cascade and complement system for SLE disease severity varied according to the levels of inflammatory reaction

To examine the extent to which the interaction effect of the coagulation cascade and complement system on SLE disease severity varied according to the levels of inflammatory response, inflammatory cytokine scores were divided into two groups by its median value (high cytokine score versus low cytokine score). Then, all of the analyses described above were repeated in the high and low cytokine score groups, respectively.

In the high cytokine score group, both coagulation score (β = 1.047, 95% CI 0.602 to 1.491, *P* < 0.001) and complement score (β = 0.437, 95% CI 0.130 to 0.744, *P* = 0.006) were significantly associated with ltSLEDAI. Moreover, an interaction effect between the coagulation score and complement score was observed (*P* < 0.001) (Table 3). Specifically, in the group of low complement score, the relationship between coagulation score and ltSLEDAI was weak; the difference in mean value of ltSLEDAI between the low with the high coagulation score group was only 0.391. This difference increased to 0.897 in the group of high complement score. Similarly, in the group of low coagulation score, the relationship between complement score and ltSLEDAI was weak; the difference in mean value of ltSLEDAI between the low with the high complement score group was only 0.359. This difference increased to 0.805 in the group of high coagulation score.

**Table 3:** Comparison of ltSLEDAI in groups of patients by high/low levels of coagulation score and complement score in the high cytokine score group

	High coagulation score	Low coagulation score	Row *P* value (across coagulation score)
High complement score			
Patients, *n*	24	11	
Complement score, mean ± SD	0.928 ± 0.274	1.119 ± 0.454	
Coagulation score, mean ± SD	0.834 ± 0.264	0.150 ± 0.169	
ltSLEDAI, mean ± SD	3.093 ± 0.317	2.196 ± 0.801	0.024^a^
Low complement score			
Patients, *n*	12	9	
Complement score, mean ± SD	0.126 ± 0.228	0.049 ± 0.200	
Coagulation score, mean ± SD	0.720 ± 0.222	0.251 ± 0.140	
ltSLEDAI, mean ± SD	2.288 ± 0.611	1.837 ± 0.715	0.618^a^
Column *P* value (across complement score)			
ltSLEDAI	0.005^a^	0.886^a^	0.004^a^

^a^Changes in ltSLEDAI are statistically compared using Tamhane’s T2(M) test. SLE, systemic lupus erythematosus; SLEDAI, SLE Disease Activity Index; ltSLEDAI, log-transformed SLEDAI.

In contrast, in the low cytokine score group, a formal interaction test between coagulation score and complement score for ltSLEDAI yielded a non-significant result (*P* = 0.406).

### Testing whether there is a direct correlation between coagulation cascades with complement system in the patients with SLE

It is of interest to note that the coagulation score and complement score were independent (r = 0.151, *P* = 0.112).

### D-dimer and C4 as the surrogates for their respective pathways in the patients with SLE

We found significant correlations between D-dimer levels with the coagulation score (r = 0.423, *P* < 0.001) and between C4 levels with the complement score (r = -0.774, *P* < 0.001). These results suggest that D-dimer and C4 may be surrogates for their respective pathways. To demonstrate this possibility, all of the analyses described above were repeated using D-dimer in place of coagulation score and C4 in place of complement score. The results demonstrated a significant effect on ltSLEDAI by the interaction between D-dimer and C4 (Table 4). Moreover, inflammatory response subgroup analyses indicated that in the high cytokine score group, an interaction test between D-dimer and C4 for ltSLEDAI yielded a statistically significant result (*P* = 0.001), while in the low cytokine score group, an interaction test yielded a non-significant result (*P* = 0.738).

**Table 4:** Comparison of ltSLEDAI in groups of patients by high/low levels of D-dimer and C4

	High D-dimer	Low D-dimer	Row *P* value (across D-dimer)
Low C4			
Patients, n	31	21	
C4, mean ± SD	0.149 ± 0.072	0.169 ± 0.082	
D-dimer, mean ± SD	0.774 ± 0.060	0.581 ± 0.132	
ltSLEDAI, mean ± SD	2.768 ± 0.673	2.169 ± 0.843	0.007^a^
High C4			
Patients, n	25	35	
C4, mean ± SD	0.563 ± 0.185	0.524 ± 0.181	
D-dimer, mean ± SD	0.774 ± 0.056	0.567 ± 0.135	
ltSLEDAI, mean ± SD	1.907 ± 0.722	1.762 ± 0.839	0.475^a^
Column *P* value (across C4)			
ltSLEDAI	0.001^a^	0.059^a^	0.001^a^

^a^Changes in ltSLEDAI are statistically compared using LSD test. SLE, systemic lupus erythematosus; SLEDAI, SLE disease activity index; ltSLEDAI, log-transformed SLEDAI.

### Determination of the interaction effect between D-dimer and C4 for the risk of lupus activity

**Figure 4:**
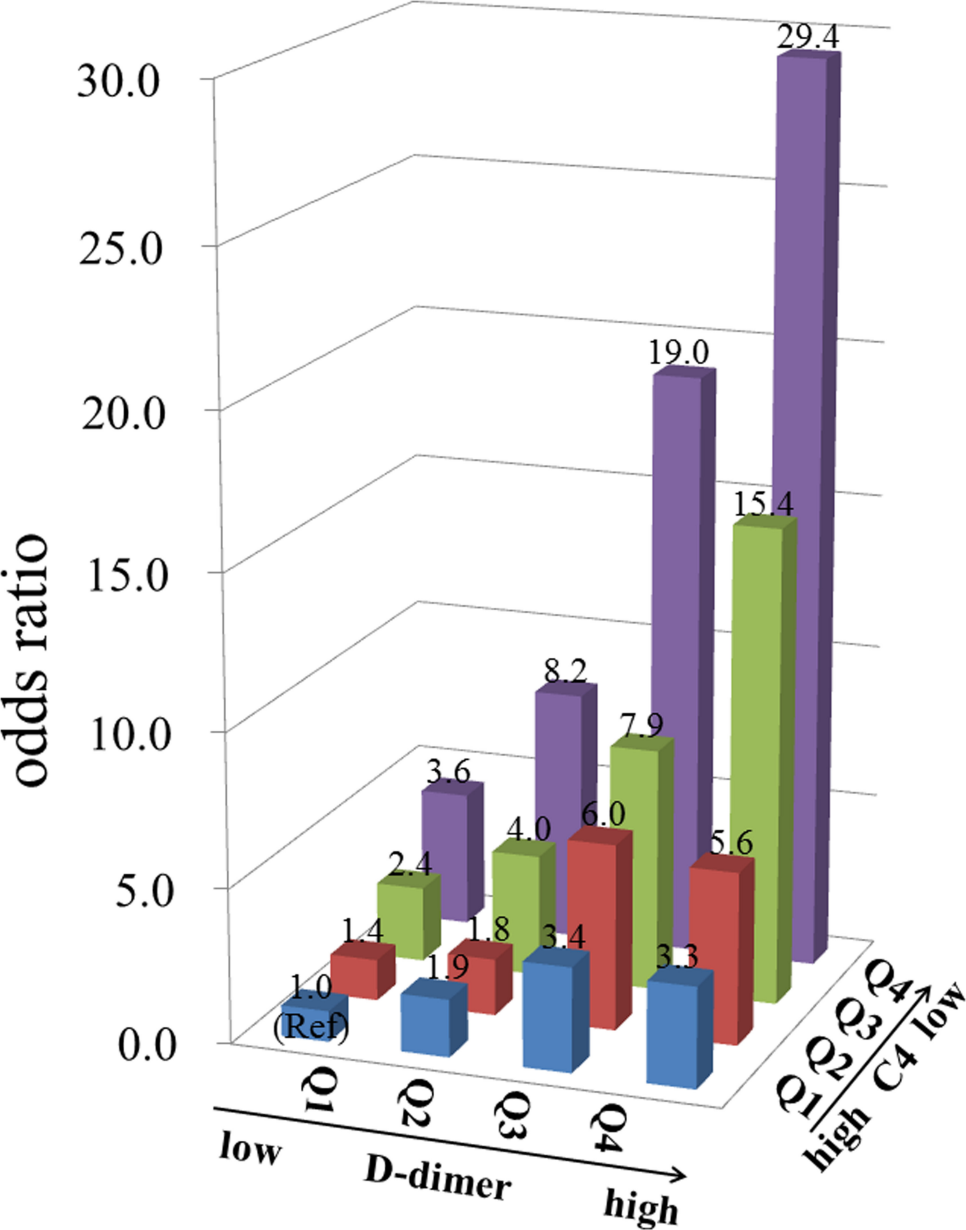
The odds ratio for lupus activity in each group according to the quartiles (Q1-Q4) of D-dimer and C4 in 2025 systemic lupus erythematosus patients Clinical data were collected from the medical records of patients hospitalized with systemic lupus erythematosus (*n* = 2025). The participants were classified into 4 categories according to the quartile of the D-dimer concentration (D-dimer ≤ 0.56, 0.56 < D-dimer ≤ 1.20, 1.20 < D-dimer ≤ 2.80, and D-dimer > 2.80 ug/mL) and further classified into 4 groups according to the quartile of the C4 concentration (C4 > 0.20, 0.12 < C4 ≤ 0.20, 0.05 < C4 ≤ 0.12 and C4 ≤ 0.05 mg/mL), with reference to the group with D-dimer ≤ 0.56 ug/mL and C4 > 0.20 mg/mL. Active lupus disease was defined as systemic lupus erythematosus disease activity index score ≥ 8.

We test whether there is an interaction effect between D-dimer and C4 for the risk of lupus activity in lupus cohort III by using a logistic regression model. The results indicated that the interaction was significant (*P* < 0.001). The odds ratios (ORs) value of the quartiles of D-dimer increased with increases in C4 categories. Similarly, the OR value of the quartiles of C4 increased with increases in D-dimer categories (Figure 4).

### Comparison of the diagnostic performance of a combination of D-dimer and C4 with that of other hematological biomarkers

**Figure 5:**
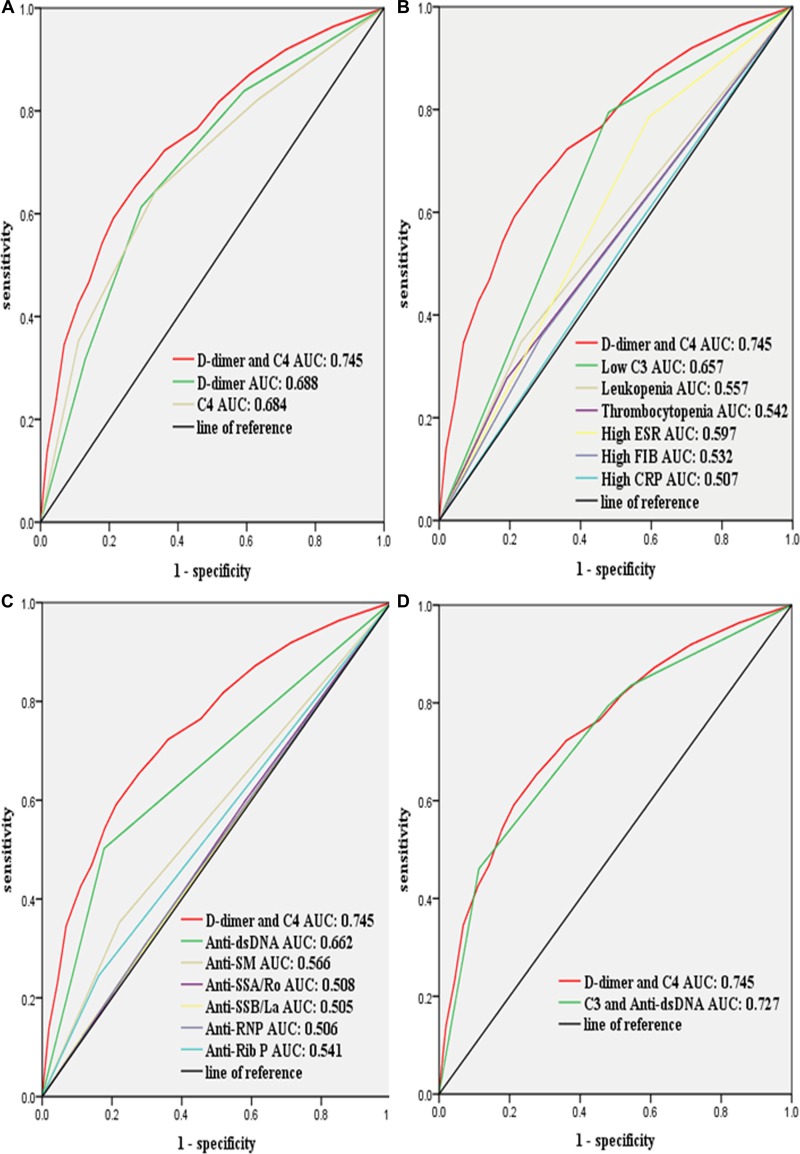
Compared the diagnostic performance of a combination of D-dimer and C4 with that of the biomarkers of hematological abnormalities for lupus activity by using receiver operating characteristic curves Clinical data were collected from the medical records of patients hospitalized with systemic lupus erythematosus (*n* = 2025). (**A**) D-dimer combined with C4 provided better diagnostic performance than D-dimer or C4 alone; (**B**) D-dimer combined with C4 provided better diagnostic performance than low C3, leukopenia, thrombocytopenia, high ESR, high FIB, or high CRP alone; (**C**) D-dimer combined with C4 provided better diagnostic performance than anti-dsDNA, anti-Sm, anti-SSA/Ro, anti-SSB/La, anti-RNP or anti-Rib P alone; (**D**) D-dimer combined with C4 provided better diagnostic performance than a combination of C3 and anti-dsDNA. Low C3 is defined as < 0.85 mg/mL; high FIB is defined as > 4.00 ug/mL; high ESR is defined as > 20.00 mm/h; high CRP is defined as > 8.00 mg/L. FIB, fibrinogen; ESR, erythrocyte sedimentation rate; CRP, C-reactive protein. Lupus activity was defined as systemic lupus erythematosus disease activity index score ≥ 8.

The area under receiver operating characteristic curves (AUROCs) was used to evaluate the diagnostic performance of D-dimer, C4 and their combination. As expected, a combination of D-dimer and C4 had better AUROCs than individual biomarkers (Figure 5A). In addition, we compared the diagnostic performance of a combination of D-dimer and C4 with that of other rhematological biomarkers. The results have shown that a combination of D-dimer and C4 provided better diagnostic performance than all individual biomarkers (Figure 5B and Figure 5C). Of these individual biomarkers, C3 and anti-dsDNA had better AUROCs than others. We then compared the diagnostic performance of a combination of D-dimer and C4 with that of a combination of C3 and anti-dsDNA. The results have shown that a combination of D-dimer and C4 provided better diagnostic performance (Figure 5D).

### Developing a multivariate predictive equation based on D-dimer and C4 levels to assess the likelihood of lupus activity

A predictive equation for lupus activity was created: P = 1 / (1 + e^−z^), where z = -1.612 + 0.361 (D-dimer) + 0.325 (C4) + 0.093 (interaction). For example, if a patient had a D-dimer level of > 2.80 ug/mL and C4 level of ≤ 0.05 mg/mL, the probability of lupus activity could be computed as follows: z = -1.612 + 0.361 × 4 + 0.325 × 4 + 0.093 × 4 × 4 = 2.620. The probability was therefore given by P = 1 / (1 + e^-2.620^) = 0.932. The performance of the predictive equation depended on the cut-off used to define a positive test and the accuracy of the predictive equation was summarized in Table 5.

**Table 5:** Sensitivity, specificity, predictive values and Youden index of the predictive equation according to different cut-offs for the diagnosis of lupus activity in 2025 SLE patients

*P* (cut-off)	Variable	Sensibility	Specificity	PPV	NPV	Youden index
0.932	D-dimer > 2.80 ug/mL C4 ≤ 0.05 mg/mL	0.138	0.981	0.927	0.389	1.119
0.868	D-dimer > 1.20 ug/mL C4 ≤ 0.05 mg/mL	0.253	0.956	0.911	0.417	1.209
0.760	D-dimer > 0.56 ug/mL C4 ≤ 0.05 mg/mL	0.327	0.919	0.878	0.433	1.246
0.604	D-dimer > 0 ug/mL C4 ≤ 0.05 mg/mL	0.352	0.890	0.851	0.435	1.242
0.872	D-dimer > 2.80 ug/mL C4 ≤ 0.12 mg/mL	0.231	0.956	0.904	0.411	1.187
0.773	D-dimer > 2.80 ug/mL C4 ≤ 0.20 mg/mL	0.274	0.924	0.866	0.416	1.199
0.629	D-dimer > 2.80 ug/mL C4 > 0 mg/mL	0.318	0.869	0.813	0.417	1.188
0.783	D-dimer > 1.20 ug/mL C4 ≤ 0.12 mg/mL	0.425	0.890	0.873	0.464	1.315
0.655	D-dimer > 0.56 ug/mL C4 ≤ 0.12 mg/mL	0.562	0.788	0.826	0.502	1.350
0.501	D-dimer > 0 ug/mL C4 ≤ 0.12 mg/mL	0.642	0.667	0.775	0.511	1.309
0.664	D-dimer > 1.20 ug/mL C4 ≤ 0.20 mg/mL	0.517	0.825	0.841	0.489	1.342
0.519	D-dimer > 1.20 ug/mL C4 > 0 mg/mL	0.613	0.707	0.789	0.506	1.320
0.533	D-dimer > 0.56 ug/mL C4 ≤ 0.20 mg/mL	0.696	0.629	0.770	0.536	1.324
0.398	D-dimer > 0 ug/mL C4 ≤ 0.20 mg/mL	0.820	0.370	0.699	0.536	1.191
0.406	D-dimer > 0.56 ug/mL C4 > 0 mg/mL	0.839	0.407	0.716	0.586	1.246
0.303	D-dimer > 0 ug/mL C4 > 0 mg/mL	1.000	0.000	0.641	0.000	1.000

SLE, systemic lupus erythematosus; P, probability; PPV, positive predictive value; NPV, negative predictive value.

## DISCUSSION

Numerous studies have investigated the levels of complement and coagulation-related molecules in the patients with SLE [2, 3]. The results indicated that dysregulation of complement and coagulation-related molecule has a role in pathogenic process of SLE. However, coordinated changes of complement and coagulation-related molecules have not been analyzed as usually each molecule is analyzed independently by specific immunoassays. With the background of such a wide range of complement and coagulation-related molecules having an implication in pathogenic process of SLE, omics techniques have revealed as a very useful tool for the evaluation of global systemic changes in molecule expression [13, 39]. To this end, transcriptomics, proteomics and metabolomics have been used recently to define molecule expression signatures in patients with SLE. These studies have demonstrated pathogenic roles of several key signaling pathways for the development of SLE, including coagulation cascade and complement system [5–8, 40–47]. However, a common limitation in these studies is that the experiment was usually performed by single omics technology. Recently, many researchers have utilized multiple omics technologies in a certain experiment that is an integrated approach able to give a much more detailed view of signaling pathways than when used individually [10–13, 48–50]. However, to date no study focused on a combined analysis in SLE. In this study, we performed the transcriptomics, proteomics and metabolomics simultaneously in the patients with SLE and HCs. The results revealed significant alterations of many of the components of the coagulation cascade and complement system in SLE at the transcriptional, proteomic and metabolic levels, the majority of which were consistent with previous immunoassays results [2, 3]. To gain deeper insights into coagulation cascade and complement system, a combined mapping of transcriptomic, proteomic and metabolic data to these pathways was performed. This analysis revealed the common links for the components of coagulation cascade and complement system at the gene, protein and metabolite level. It is interesting to note that (i) varying or even contradicting data correlations between transcriptome and proteome have been found, such as SERPING1 secretion that is independent from gene transcription. Thus, the function of the coagulation cascade and complement system may result from the concerted action of gene and protein ensembles, rather than from the isolated action of single players; (ii) a pathway map exhibits various possible routes that one can take. For example, the dysregulation of metabolite Frbrin can be caused by the abnormalities of F13 or FIB or a combination of these factors. Our failure to produce effective therapeutic drugs could, in part, be related to our lack of a deeper understanding of all possible routes that one can take [51, 52]. Altogether, an integrated omics approach has the potential to considerably advance our understanding of the coagulation cascade and complement system in SLE.

Although coagulation cascade and complement system are two distinct multi-component networks, numerous studies have independently reported several interesting interactions between these two pathways and the contribution of these interactions to the development of various clinical conditions [14, 18–20]. Consistently, we found that coagulation cascade and complement system had an interaction effect on SLE disease severity. Mechanically, previous studies have shown that the components of coagulation cascade and complement system can interact with each other directly, leading to the activation of both pathways simultaneously [15–17]. However, no significant correlation was observed between coagulation cascade and complement system in this study, suggesting the direct interaction between these two pathways is disease-specific and may not occur in the patients with SLE. Recently, evidence from *in vitro* and *in vivo* models is accumulating to support the indirect interactions between coagulation cascades and complement system through the involvement of inflammatory mediators [18–22]. In the present study, the interaction effect between the coagulation cascade and complement system on SLE disease activity was pronounced among patient with excess inflammatory cytokines. Drawing together several lines of evidence that (i) SLE is a systemic autoimmune disease, characterized by excess inflammatory cytokine production [1]; (ii) the extent of complement cleavage in SLE depends in part on the levels of inflammatory response [53–55]; (iii) inflammatory cytokines facilitate coagulation factor activation in SLE [56], and (iv) inflammatory mediators can serve as bridge between coagulation cascade and complement system [18–22], an attractive mechanism arises that during an inflammatory response, the reactions of coagulation cascade and complement system occurred not only coincidentally but also in a self-reinforcing manner in patients with SLE that lead to exacerbation of the disease.

SLEDAI is considered as the ‘gold standard’ for the diagnosis of lupus activity [23]. However, this tool is apparently not easy to implement. Recently, new biomarkers are emerging as research on SLE progresses [24–38]. However, these biomarkers are hard to measure, relatively expensive and not routinely assayed. Compared with these biomarkers, D-dimer and C4 are more stable and easier to be measured routinely in most hospitals. Therefore, D-dimer and C4 may serve as good biomarkers for lupus activity. Previous studies on D-dimer levels in patients with SLE mainly focused on the role of D-dimer in clinical manifestations. The importance effect of D-dimer on the development of serositis and vascular disease in patients with SLE has been confirmed [57, 58]. However, the diagnostic value of D-dimer for lupus activity has rarely been examined. With respect to C4, there is still some uncertainty about the use of this parameter as biomarker for the diagnosis of lupus activity. One study reported that serum levels of C4 are useful in disease activity evaluation in patients with SLE [59]. In contrast, another study found that serum levels of C4 split product C4d rather than C4 can be used as biomarker for lupus activity [60]. A recent study uncovered the molecular heterogeneity of SLE [61]. This suggests that a combination of several biomarkers rather than a single biomarker will be required by clinicians for lupus activity diagnosis [62]. Therefore, studies working with a combination of biomarkers that are involved in biologically relevant pathways may be more meaningful and more substantial than studies focus on single biomarkers. In the present study, we evaluated the diagnostic value of a combined D-dimer and C4 for lupus activity in a large cohort of patients with SLE. The results demonstrated that a combination of D-dimer and C4 provided good diagnostic performance. Based on D-dimer and C4 levels, a multivariate predictive equation was developed to assess the likelihood of lupus activity. As with all other prediction models [63–65], it must be made clear that such a model never predicts the specific outcome of an individual patient. However, with a predictive model, we can determine the probability of lupus activity. In clinical practice, a clinician can determine what probability of lupus activity constitutes a reasonable threshold when therapy with potentially significant side effects is being considered. If a therapy has minimal side effects and the delay in identifying patients with high disease activity inhibits the benefit of therapy, it may be reasonable to offer the therapy to patients whose probability of lupus activity falls below 60%. If a therapy has substantial risk, such as with cyclophosphamide [66], it may be reasonable to restrict it to those whose risk of lupus activity exceeds 80%.

There is some weakness in our study. First, to better correlate SLE disease severity with signaling pathway, it is ideal to collect blood samples and clinical data of SLE patients before starting DMARDs treatment. However, in reality it is challenging to obtain these samples and data. In our study, part of the blood samples and clinical data was obtained from the SLE patients who had been receiving treatment for more than a month. Second, because of the cross-sectional nature of this study, associations do not necessarily mean causality, particularly when these include potentially co-dependent variables. For instance, we cannot rule out the possibility that exacerbation of the SLE somehow causes dysfunctional coagulation cascade and complement system as well as excess inflammation, although it is difficult to postulate a potential mechanism. Similarly, we cannot exclude the possibility that excess inflammation (with accompanying lupus activity) causes the dysregulation of coagulation cascade and complement system. Prospective studies are required in the future.

In summary, this manuscript consists of three parts. In the first part (multi-omics analysis), transcriptomics, proteomics and metabolomics were performed simultaneously in the patients with SLE and HCs to identify the differentially expressed coagulation and complement-related genes ⁄ proteins ⁄ metabolites. Further analysis by integration of multi-omics data provided several novel findings as illustrated in Figure 2. First, coagulation and complement-related gene levels do not ultimately determine protein levels, such as SERPING1 concentration that is independent from gene transcription. Second, one biological reaction can be caused by different routes as shown by the fact that the dysregulation of metabolite Frbrin can be caused by the abnormalities of F13 or FIB or a combination of these factors. To our knowledge, this is the first study integrating multi-omics data sets to analyze the pathogenic signaling pathways in SLE and will serve as basis for future investigations in relevant research areas. In the second part (protein expression analysis), we define the relationships between the coagulation cascade, complement system, inflammatory response and SLE disease severity that has not been previously described. We have found that coagulation cascade and complement system have an interaction effect on SLE disease severity and this effect is pronounced among patients with excess inflammation. In the third part (clinical data analysis), we have shown for the first time a good diagnostic power of a combined D-dimer and C4 for lupus activity. Moreover, by using D-dimer and C4, we have created a novel predictive equation for lupus activity: P = 1 / (1 + e^−z^), where z = -1.612 + 0.361 (D-dimer) + 0.325 (C4) + 0.093 (interaction). This multivariate equation improves current methods for the diagnosis of lupus activity and can be easily implemented in an inexpensive programmable calculator.

## MATERIALS AND METHODS

### Subjects

The protocol for our study was consistent with the provisions of the World Medical Association Declaration of Helsinki, and informed consent was obtained from each subject before enrolment. The study was approved by the medical ethics committee of Anhui Medical University. Methods were carried out in accordance with the approved guidelines. The patients were recruited from the First Affiliated Hospital of Anhui Medical University and Anhui Provincial Hospital. All patients fulfilled at least 4 of the SLE classification criteria of the American College of Rheumatology [23]. Clinical manifestations of SLE patients, such as lupus nephritis, arthritis and skin rash, were recorded. Laboratory abnormalities, including thrombocytopenia (< 100 × 10^9^/L), leukopenia (< 4.0 × 10^9^/L), hematuria (> 5 RBC/HP), proteinuria (> 0.5 g/day), the presence of anti-dsDNA and levels of C3 and C4, were also retrieved from the medical record. SLE disease activity was evaluated by SLEDAI scores [23]. Active lupus disease was defined as SLEDAI scores ≥ 8.

### Collection of blood samples and clinical data

Blood samples were collected into different tubes. Blood samples for transcriptomics were collected in PAXgene Blood RNA tubes, allowed to remain at room temperature for 2 h, frozen at –20°C overnight, and then stored at –80°C until RNA isolation. Blood samples for proteomics and metabolomics were collected in EDTA tubes and centrifuged at 3000 rpm for 15 min at 4°C. Blood samples for measurement of serum complement and inflammatory cytokine were collected in plain tubes. Blood samples for measurement of plasma coagulation were collected in tubes containing 0.106 M trisodium citrate. All samples were aliquoted prior to storage at –80°C. Only one aliquot was retrieved for each assay to avoid multiple freeze/thaw cycles.

Clinical data were collected from consecutive patients with the following criteria: (1) diagnoses of systemic lupus erythematosus (SLE), (2) plasma coagulation D-dimer and serum complement C4 measured by routine analyses, (3) complete clinical and laboratory data available for calculation of SLE disease activity index (SLEDAI) score. We excluded any patient missing data on demographic information and medication intake because these variables were controlled for subsequent analyses. With these criteria, a final sample of 2025 patients contributed to the analyses.

### Transcriptomics (RNA-sequencing, RNA-seq)

Total RNA was isolated with PAXgene Blood RNA Kit. To minimize the biological variation, within each group [SLE patients and healthy controls (HC)], equal amounts of sample from 8 different individuals were randomly pooled. For each group, three biological replicate samples of patient (SLE_1; SLE_2; SLE_3) and control (HC_1; HC_2; HC_3) were included. The RNA was dissolved in RNase-free water (New England BioLabs) to remove residual DNA. The concentration of RNA was determined by NanoDrop 2000 (Thermo Fisher Scientific), and the RNA integrity value (RIN) was checked using 2100 Bioanalyzer (Agilent).

The cDNA libraries were prepared according to the Illumina manufacturer’s instructions. The poly(A) containing mRNA molecules were purified using Sera-mag Magnetic Oligo(dT) Beads (Illumina) from RNA of each sample. Ten milli molar Tris-HCl was used to elute the mRNA from the magnetic beads. To avoid priming bias when synthesizing cDNA, the mRNA was first fragmented before cDNA synthesis. The mRNA was fragmented into small pieces using divalent cations at elevated temperature. The cleaved mRNA fragments were converted to double-stranded cDNA using SuperScript II, RNaseH and DNA Pol I. The resulting cDNA was purified using the QIA quick PCR Purification Kit (Qiagen). Then, cDNA was subjected to end-repair and phosphorylation using T4 DNA polymerase, Klenow DNA polymerase and T4 polynucleotide kinase, and subsequent purification using QIA quick PCR Purification Kit (Qiagen). These repaired cDNA fragments were adenylated using Klenow DNA polymerase, producing cDNA fragments with a single ‘A’ base overhung at their 3’ ends for subsequent adapter ligation. Adapters were ligated to the ends of these 3’ adenylated cDNA fragments, followed by purification using MinElute PCR Purification Kit (Qiagen). To select a size range of templates for downstream enrichment, the products of the ligation reaction were purified on a 2% agarose gel (Bio-rad). A range of cDNA fragments (200 ± 25 bp) was excised from the gel and extracted using QIA quickGel Extraction Kit (Qiagen). Fifteen rounds of PCR amplification were performed to enrich the adapter modified cDNA library. The PCR products of size 200 ± 25 bp were purified using QIAquick GelExtraction Kit except that Qiaquick spincolumns were substituted with MinElute spin columns (Qiagen). Finally, after quantification on a 2100 Bioanalyzer using the DNA1000 chip kit (Agilent), the cDNA library products were sequenced using the Illumina HiSeq 2000 according to manufacturers’ protocols.

Raw reads were filtered using a fastx tool kit and then mapped to the human genome 19 (hg19). To obtain quantification scores for all genes, fragments per kilobase of exon model per million mapped reads (FPKM) values were calculated by using the RSEM program [67]. We used the NOISeq package to identify differentially expressed genes [68]. Probability ≥ 0.8 and an absolute value of log2Ratio ≥ 1 were used as the threshold to judge the differentially expressed genes. The filtered clean read transcriptomics data have been deposited to the National Center for Biotechnology Information under accession number SRP076773.

### Proteomics (isobaric tags for relative and absolute quantitation, iTRAQ)

To reduce the complexity of samples, the highly abundant proteins were depleted using ProteoMinerTM Kits (Bio-Rad) according to the manufacturer’s protocol. Samples were eluted in Lysis buffer (7 M Urea, 2 M Thiourea, 4% 3-[(3-Cholamidopropyl)dimethylammonio]propanesulfonate, 40 mM Tris-HCl, pH 8.5) and reduced with 10 mM dithiothreitol at 56°C for 1 h, followed by alkylation with 55 mM iodoacetamide (IAM) in the darkroom for 1 h. The reduced and alkylated protein mixtures were precipitated by adding chilled acetone at -20°C overnight. After centrifugation, the pellet was dissolved in 0.5 M tetraethyl-ammonium bromide (TEAB) (Applied Biosystems) and sonicated in ice. An aliquot of the supernatant was then taken for determination of protein concentration by the Bradford assay. The proteins in the supernatant were kept at -80°C for further analysis.

Total protein (100 μg) was taken out of each sample solution. Then, the protein was digested with Trypsin Gold (Promega) with the ratio of protein : trypsin = 30:1 at 37°C for 16 h. After trypsin digestion, peptides were dried by vacuum centrifugation. Peptides were reconstituted in 0.5 M TEAB and processed according to the manufacture’s protocol for 8-plex iTRAQ reagent (Applied Biosystems). Briefly, one unit of iTRAQ reagent was thawed and reconstituted in 24 μl isopropanol. Samples were labeled with the iTRAQ tags as follow: SLE_1, isobaric tags 113; SLE_2, isobaric tags 114; SLE_3, isobaric tags 119; HC_1, isobaric tags 116; HC_2, isobaric tags 117 and HC_3, isobaric tags 118. The protocols which generate such biological replicate samples were the same as that in transcriptomics analysis. The labeled peptides were incubated at room temperature for 2 h and then mixed and dried by vacuum centrifugation.

Strong cation exchanger (SCX) chromatography was performed with a LC-20AB high performance liquid chromatography (HPLC) pump system (Shimadzu). The iTRAQ-labeled peptide mixtures were reconstituted with 4 ml buffer A [25 mM NaH2PO4 in 25% acetonitrile (ACN), pH 2.7] and loaded onto a 4.6×250 mm SCX column containing 5 μm particles (Phenomenex). The peptides were eluted at a flow rate of 1 ml/min with a gradient of buffer A for 10 min, 5 - 60% buffer B [25 mM NaH2PO4, 1 M kalium chloratum (KCl) in 25% ACN, pH 2.7] for 27 min, 60–100% buffer B for 1 min. The system was then maintained at 100% buffer B for 1 min before equilibrating with buffer A for 10 min prior to the next injection. Elution was monitored by measuring the absorbance at 214 nm, and fractions were collected every 1 min. The eluted peptides were pooled into 20 fractions, desalted with a Strata X C18 column (Phenomenex) and vacuum-dried.

For each biological replicate, three technical repeats (T1; T2; T3) were processed. Each fraction was resuspended in buffer A (2% ACN, 0.1% formic acid) and centrifuged at 20000 rpm for 10 min. The final concentration of peptide was 0.5 ug/ul. Then, 10 μl supernatant was loaded on a LC-20AD nanoHPLC (Shimadzu). The peptides were eluted onto a 10 cm analytical C18 column. The samples were loaded at 8 μl/min for 4 min, then the 44 min gradient was run at 300 nl/min starting from 2 to 35% buffer B (98% ACN, 0.1% FA), followed by 2 min linear gradient to 80%, maintenance at 80% buffer B for 4 min, and finally return to 5% in 1 min.

The peptides were subjected to nanoelectrospray ionization, followed by tandem mass spectrometry (MS/MS) in a Q-Exactive orbitrap (Thermo Fisher Scientific) coupled online to the HPLC. Intact peptides were detected in the orbitrap at a resolution of 70000. Peptides were selected for MS/MS using high-energy collision dissociation (HCD) operating mode. Ion fragments were detected in the orbitrap at a resolution of 17500. A data-dependent procedure that alternated between one MS scan followed by fifteen MS/MS scans was applied for the 15 most abundant precursor ions. The electrospray voltage was 1.6 kV. Automatic gain control (AGC) was used to optimize the spectra generated by the orbitrap. The AGC targets for MS and for MS/MS were 3e6 and 1e5, respectively. For MS scans, the m/z scan range was 350 to 2000 Da. For MS/MS scans, the m/z scan range was 100 to 1800 Da.

Raw data files acquired from the orbitrap were converted into MGF files using Proteome Discoverer 1.2 (Thermo Fisher Scientific). Protein identification was performed by Mascot search engine (version 2.3.02) against the database of Uniprot-proteome-homo-sapiens-9606_20150316 (68015 sequences) [69]. For protein identification, a mass tolerance of 20 ppm was permitted for intact peptide masses and 0.05 Da for fragmented ions. The potential variable modifications were Gln->pyro-Glu (N-term Q), Oxidation (M) and Deamidated (NQ). The fixed modifications were Carbamidomethyl (C), iTRAQ-8plex (N-term) and iTRAQ-8plex (K). The charge states of peptides were set to +2 and +3. To reduce the probability of false peptide identification, peptides obtained after applying 1% false discovery rate (FDR) cut off were selected for further analysis. Each confident protein contains at least one unique peptide. For protein quantitation, it was required that a protein contains at least two unique spectra. The quantitative protein ratios were normalized by the median peptide ratio in Mascot. The differentially expressed proteins have been considered as significant if they met the following criteria: (1) fold change of ratio > 1.20 or < 0.83 and *P*-value < 0.05, (2) satisfied the first criteria in at least 2 of 3 technical replicates. Proteins that met these criteria were excluded if discordant trend in expression had emerged within the three technical replicates. The final fold change of protein was calculated as the average value obtained from all technical replicates. The mass spectrometry proteomics data have been deposited to the ProteomeExchange database under accession number PXD004443 through the PRIDE website (http://www.ebi.ac.uk/pride/). Username: reviewer69314@ebi.ac.uk, Password: 4rzbNxdo.

### Metabolomics

Plasma samples (50 ul) were thawed and then precipitated by 200 μl methanol. After centrifugation at 14000 rpm for 10 min at 4°C, 10 μl supernatant was transferred into the HPLC-MS. A “quality control” (QC) sample was also prepared by mixing equal volumes (10 μl) from each plasma sample before sample preparation.

LC-MS data were acquired using HPLC system (Shimadzu) coupled online to a LTQ orbitrap velos instrument (Thermo Fisher Scientific). Sample analysis was carried out in positive ion modes. The mass scanning range was 50–1000 m/z and the capillary temperature was 350°C. Nitrogen sheath gas was set at a flow rate of 30 l/min. Nitrogen auxiliary gas was set at a flow rate of 10 l/min. Spray voltage was set to 4.5 kV. The LC-MS system was run in binary gradient mode. Solvent A was 0.1% formic acid/water, and solvent B was 0.1% formic acid/methanol. The flow rate was 0.2 ml/min. A C-18 column (150 mm×2.1 mm, 3.5 um) (Agilent) was used for all analysis. The gradient was as follows: 5% solvent B at 0 min, 5% solvent B at 5 min, 100% solvent B at 8 min, 100% solvent B at 9 min, 5% solvent B at 18 min, and 5% solvent B at 20 min. The pooled “QC” sample was injected five times at the beginning of the run to ensure system equilibrium and then every 5 samples to further monitor the stability of the analysis [70, 71].

The acquired MS data pretreatments, including peak picking, peak grouping, retention time correction, second peak grouping and annotation of isotopes and adducts, were achieved using the XCMS and CAMERA software. LC-MS raw data files were initially converted into mzXML format by ReAdW software (version 4.3.1), then directly processed by the XCMS and CAMERA software. A list of the ion intensities of each peak was generated using retention time and the m/z data pairs as identifiers for each ion. The resulting three-dimensional matrix contained peak indices (retention time-m/z pairs), sample names (observations) and ion intensity information (variables). To obtain consistent variables, the resulting matrix was further reduced by removing peaks with more than 80% missing values. To ensure that data are of comparable high quality within an analytical run, an approach based on the periodic analysis of a standard biological quality control sample (QC sample) together with the true samples is now accepted as a quality assurance strategy in metabolic profiling. Here, each retained peak is normalized to the QC sample using robust loess signal correction (R-LSC). A threshold of 30% was set for the relative standard deviation (RSD) values of metabolites in the QC samples, which is accepted as a standard in the assessment of repeatability in metabolomics data sets. Finally, the filtered matrix was exported for multivariate statistical analysis using partial least-squares discriminant analysis (PLS-DA). For each metabolite peak, Mann−whitney−wilcoxon test was applied to measure the significance of each metabolite among the different trimesters, with results adjusted for multiple testing using FDR correction. On the basis of a variable importance in the projection (VIP) threshold > 1, a number of metabolites responsible for the difference in the metabolic profile scan of three trimesters can be obtained. The metabolites identified by two latent variables of the PLS-DA model were validated at a univariate level using FDR test with the critical *P* value set to not higher than 0.05.

Exact molecular mass data from significant peaks were used to search the databases, such as HMDB, BioCyc, KEGG, LIPID as well as MetaCyc, for metabolite identities. A metabolite name was reported when a mass difference between observed and theoretical mass was < 20 ppm. Isotopic distribution measurements were used to further validate the molecular formula of matched metabolites. The identities of key metabolites were confirmed by comparison of their MS/MS spectra and retention time with those obtained using 9 available reference standards in our laboratory. The mass spectrometry metabolomics data have been deposited to the public repository MassIVE under accession number MSV000079834, which can be found in ftp://MSV000079834@massive.ucsd.edu. Username: MSV000079834, Password: a.

### Protein expression analysis

We measured the levels of seven serum complements, ten plasma coagulations and three serum inflammatory cytokines by commercially available ELISA kits. The serum complements included C3a (Catalog No. 550499, BD), C4a (Catalog No. 550947, BD), C5a (Catalog No. 557965, BD), mannose-binding lectin-associated serine proteinase 2 (MASP2) (Catalog No. HK326, Hycult Biotech), C1q (Catalog No. ab170246, Abcam), C7 (Catalog No. ab125964, Abcam) and factor I (FI) (Catalog No. ab195460, Abcam). The plasma coagulations included factor 7 (F7) (Catalog No. ab108829, Abcam), factor 9 (F9) (Catalog No. ab108831, Abcam), factor 12 (F12) (Catalog No. ab108835, Abcam), factor 13 (F13) (Catalog No. ab108836, Abcam), fibrinogen (FIB) (Catalog No. ab108842, Abcam), thrombin-antithrombin complex (TAT) (Catalog No. ab108907, Abcam), Von Willebrand factor (VWF) (Catalog No. ab108918, Abcam), protein S (PROS) (Catalog No. ab125969, Abcam), D-dimer (Catalog No. ab196269, Abcam) and antithrombin-III (ATIII). (Catalog No. DSPC10, RD). The serum inflammatory cytokines included tumor necrosis factor-receptor II (TNF-RII) (Catalog No. DRT200, RD), interleukin (IL)-6 (Catalog No. 430507, BioLegend) and IL-8 (Catalog No. D8000C, RD). In addition, the level of serum C4 was measured in the clinical laboratory using nephelometric method.

We divided the 21 analytes mentioned above into two groups: the pathway activators, including C3a, C4a, C5a, MASP2 and C7 (complement pathway), VWF, F7, TAT, FIB, F9 and D-dimer (coagulation pathway) as well as TNF-RII, IL-6 and IL-8 (inflammatory responses), and pathway inhibitors or de-activators, including C1q, FI and C4 (complement pathway) as well as PROS, F12, F13 and ATIII (coagulation pathway). Two approaches were used to achieve this: one use data from our proteomics experiment and another was based on the published literatures [1, 2]. The pathway activators were significantly elevated in SLE patients and their levels were positively correlated with the level of the activation of the pathway. In contrast, the pathway inhibitors or de-activators were significantly decreased and their levels were negatively associated with the level of the activation of the pathway. The summary complement score was based on the levels of C3a, C4a, C5a, MASP2, C7, C1q, FI and C4. When we calculated the summary complement score, pathway activators were regarded as positive number while pathway inhibitors or de-activators were viewed as negative number. Concentration values were normalized across all samples so that the maximum value for any analytes was 1. Values for each sample were then summed to derive the final score. The summary coagulation score and cytokine score were calculated for each sample in a way similar to the calculation of the summary complement score.

### Statistical analysis

For the multi-omics analysis, GO is an international standardization of gene function classification system. Used for the bioinformatic analysis, it provides a set of dynamic updating controlled vocabulary to describe genes and gene products attributes in the organism. GO has three ontologies, which can describe molecular function, cellular component and biological process. KEGG pathway is a collection of manually drawn pathway maps representing our knowledge on the molecular interaction and reaction networks. Heatmap was constructed using ggplot2 of R software (version 3.1.3).

For the protein expression analysis, we assessed the principal effects of coagulation score and complement score on ltSLEDAI by using multivariate linear regressions. The results are presented as β-coefficient along with their 95% confidence intervals. The interaction effect was assessed as a test of a product term formed from the coagulation score and complement score. To help understand the interaction effect, we dichotomized the coagulation score and complement score into two groups, respectively (low coagulation score versus high coagulation score; low complement score versus high complement score). With these criteria, participants were then categorized as: (1) low coagulation score and low complement score (as reference group in this analyses), (2) low coagulation score and high complement score, (3) high coagulation score and low complement score, and (4) high coagulation score and high complement score. Multiple group comparisons were performed, and significant differences among groups were assessed by LSD test assuming equal variances or Tamhane’s T2(M) test assuming unequal variances.

We next examined the extent to which the interaction effect of the coagulation cascade and complement system on SLE disease severity varied according to the levels of inflammatory reaction. For this analysis, cytokine scores were divided into two groups by its median value (low cytokine score versus high cytokine score). All of the analyses described in the above paragraph were repeated in the high and low cytokine score groups, respectively.

To assess whether D-dimer and C4 could be surrogates for their respective pathways, the above analyses were repeated using D-dimer in place of coagulation score and C4 in place of complement score.

For the clinical data analyses, we used a logistic regression model to test the possible interaction between D-dimer and C4 on the risk for lupus activity. Results are presented as ORs along with their 95% confidence intervals. For this analysis, the participants were classified into four categories according to the quartile of D-dimer concentration (D-dimer ≤ 0.56, 0.56 < D-dimer ≤ 1.20, 1.20 < D-dimer ≤ 2.80, and D-dimer > 2.80 ug/mL) and further classified into 4 groups according to the quartile of C4 concentration (C4 > 0.20, 0.12 < C4 ≤ 0.20, 0.05 < C4 ≤ 0.12 and C4 ≤ 0.05 mg/mL), with reference to the group with D-dimer ≤ 0.56 ug/mL with C4 > 0.20 mg/mL. All participants were assigned to one of 16 categories.

The diagnostic values of D-dimer, C4 and other hematological biomarkers were assessed by calculating AUROCs.

Based on D-dimer and C4 levels, a predictive equation was developed to assess the likelihood of lupus activity. The probability of lupus activity in an individual patient was derived from the following formula: P = 1 / (1 + e^−z^), where *P* is the probability of lupus activity and z = β_0_ (derived constant) + β_1_ (D-dimer) + β_2_ (C4) + β_3_ (interaction). For a range of probabilities that might be used to identify individuals with lupus activity, the accuracy of the predictive equation was evaluated by calculating sensitivity, specificity, positive and negative predictive values, and Youden index.

Categorical variables were reported as frequencies and proportions. Continuous variables were reported as mean ± SD or medians with range, depending on their distribution. The Pearson correlation coefficient was used for correlation analyses. *P* value < 0.05 was considered statistically significant. All statistical analyses were performed using SPSS 13.0 (Chicago, Illinois, USA).

## SUPPLEMENTARY MATERIALS TABLES















## Abbreviations

SLE: systemic lupus erythematosus
C5: complement 5
TNF-α: tumor necrosis factor
IL-6: interleukin-6
DMARDs: disease modifying anti-rheumatic drugs
SLEDAI: SLE disease activity index
TIM3: T cell immunoglobulin domain and mucin-3
HCs: healthy controls
GO: gene ontology
KEGG: kyoto encyclopedia of genes and genomes
iTRAQ: isobaric tag for relative and absolute quantitation
MASP2: mannan-binding lectin-associated serine protease 2
VWF: Von Willebrand factor
PROS: protein S
ATIII: antithrombin-III
TAT: thrombin-antithrombin complex
AUROCs: area under receiver operating characteristic curves
