# Supplementary material for: Coagulation cascade and complement system in systemic lupus erythematosus
Source: Oncotarget. 2017 Dec 11;9(19):14862–81. doi: 10.18632/oncotarget.23206 (PMC5871083; doi:10.18632/oncotarget.23206)
Supplement: Supplementary file 1 [file oncotarget-09-14862-s001.pdf]

Coagulation cascade and complement system in systemic lupus erythematosus

SUPPLEMENTARY MATERIALS

Supplementary Table 1: Identification of genes that are differentially displayed in SLE patients using RNA-sequencing. See_Supplementary_Table 1

Supplementary Table 2: Selected GO biological processes enrichments identified among differentially expressed genes in SLE patients. See_Supplementary_Table 2

Supplementary Table 3: Selected KEGG pathway enrichments identified among DEGs in SLE patients

Pathway	DEGs with pathway annotation (326)	All genes with pathway annotation (17252)	*p*-value	Pathway ID
Hematopoietic cell lineage	18 (5.52%)	152 (0.88%)	0.000	ko04640
Systemic lupus erythematosus	17 (5.21%)	188 (1.09%)	0.000	ko05322
Asthma	9 (2.76%)	47 (0.27%)	0.000	ko05310
Leishmaniasis	12 (3.68%)	113 (0.65%)	0.000	ko05140
Measles	13 (3.99%)	188 (1.09%)	0.000	ko05162
Staphylococcus aureus infection	11 (3.37%)	139 (0.81%)	0.000	ko05150
Malaria	8 (2.45%)	76 (0.44%)	0.000	ko05144
T cell receptor signaling pathway	12 (3.68%)	171 (0.99%)	0.000	ko04660
Cell adhesion molecules (CAMs)	13 (3.99%)	204 (1.18%)	0.000	ko04514
Osteoclast differentiation	13 (3.99%)	213 (1.23%)	0.000	ko04380
Herpes simplex infection	15 (4.6%)	285 (1.65%)	0.000	ko05168
Allograft rejection	6 (1.84%)	55 (0.32%)	0.001	ko05330
Hepatitis C	11 (3.37%)	178 (1.03%)	0.001	ko05160
Intestinal immune network for IgA production	6 (1.84%)	59 (0.34%)	0.001	ko04672
Natural killer cell mediated cytotoxicity	12 (3.68%)	218 (1.26%)	0.001	ko04650
Primary immunodeficiency	6 (1.84%)	61 (0.35%)	0.001	ko05340
NF-kappa B signaling pathway	10 (3.07%)	173 (1%)	0.002	ko04064
Autoimmune thyroid disease	6 (1.84%)	75 (0.43%)	0.003	ko05320
African trypanosomiasis	5 (1.53%)	54 (0.31%)	0.003	ko05143
HTLV-I infection	15 (4.6%)	376 (2.18%)	0.005	ko05166
Phagosome	14 (4.29%)	351 (2.03%)	0.007	ko04145
Influenza A	13 (3.99%)	328 (1.9%)	0.010	ko05164
Graft-versus-host disease	5 (1.53%)	74 (0.43%)	0.013	ko05332
Adherens junction	8 (2.45%)	173 (1%)	0.017	ko04520
Leukocyte transendothelial migration	11 (3.37%)	280 (1.62%)	0.018	ko04670
Complement and coagulation cascades	8 (2.45%)	181 (1.05%)	0.022	ko04610
Vitamin digestion and absorption	4 (1.23%)	57 (0.33%)	0.023	ko04977
B cell receptor signaling pathway	7 (2.15%)	151 (0.88%)	0.025	ko04662
Amoebiasis	9 (2.76%)	222 (1.29%)	0.026	ko05146
Antigen processing and presentation	6 (1.84%)	122 (0.71%)	0.028	ko04612

DEGs, differentially expressed genes; SLE, systemic lupus erythematosus.

Supplementary Table 4: Identification of proteins that are differentially displayed in SLE patients using iTRAQ. See_Supplementary_Table 4

Supplementary Table 5: Selected GO biological processes enrichments identified among differentially expressed proteins in SLE patients. See_Supplementary_Table 5

Supplementary Table 6: Selected KEGG pathway enrichments identified among differentially expressed proteins in SLE patients. See_Supplementary_Table 6

Supplementary Table 7: Identification of main metabolites that are differentially displayed in SLE patients

Name	Fold change	p-value	VIP	Database
tyramine	1.545	0.000	1.832	BioCyc
menadione	1.538	0.001	1.080	BioCyc
L-Tryptophan	1.629	0.000	1.936	BioCyc
Valproic acid	1.570	0.000	2.099	HMDB; KEGG; LIPID
1-alpha-25-Dihydroxyvitamin	0.545	0.005	1.788	KEGG
Linoleic acid	2.654	0.000	2.757	HMDB; KEGG; LIPID
L-leucine	1.692	0.000	2.056	BioCyc
L-isoleucine	1.692	0.000	2.056	BioCyc
2-phenylethylamine	2.354	0.000	2.422	BioCyc
Fibrin	1.772	0.000	2.207	KEGG

Metabolites that verified by reference standards are marked with underline. SLE, systemic lupus erythematosus.

Supplementary Table 8: KEGG pathway enrichments identified among differentially expressed metabolites in SLE patients. See_Supplementary_Table 8
