# Supplementary material for: Coagulation cascade and complement system in systemic lupus erythematosus
Source: Oncotarget. 2017 Dec 11;9(19):14862–81. doi: 10.18632/oncotarget.23206 (PMC5871083; doi:10.18632/oncotarget.23206)
Supplement: Supplementary file 2 [file oncotarget-09-14862-s002.docx]

**Supplementary Table 1. Identification of genes that are differentially displayed in SLE patients using RNA-sequencing.**

| **Gene ID** | **Means-HC**  **(FPKM)** | **Means-SLE**  **(FPKM)** | **Fold**  **change** | **Up/Down** | **Probability** | **Symbol** |
| --- | --- | --- | --- | --- | --- | --- |
| 153527 | 30.400 | 72.210 | 1.248 | Up | 0.850 | ZMAT2 |
| 147700 | 3.483 | 8.693 | 1.319 | Up | 0.801 | KLC3 |
| 7634 | 4.250 | 11.723 | 1.464 | Up | 0.832 | ZNF80 |
| 710 | 10.527 | 46.273 | 2.136 | Up | 0.911 | SERPING1 |
| 6513 | 5.717 | 12.767 | 1.159 | Up | 0.801 | SLC2A1 |
| 7111 | 14.400 | 33.610 | 1.223 | Up | 0.839 | TMOD1 |
| 6947 | 2.543 | 11.327 | 2.155 | Up | 0.882 | TCN1 |
| 91543 | 11.537 | 71.883 | 2.639 | Up | 0.935 | RSAD2 |
| 1088 | 3.913 | 14.690 | 1.908 | Up | 0.875 | CEACAM8 |
| 349152 | 7.607 | 20.093 | 1.401 | Up | 0.846 | DPY19L2P2 |
| 7027 | 9.487 | 26.727 | 1.494 | Up | 0.859 | TFDP1 |
| 9636 | 28.637 | 214.747 | 2.907 | Up | 0.946 | ISG15 |
| 10346 | 49.957 | 103.513 | 1.051 | Up | 0.825 | TRIM22 |
| 1669 | 0.883 | 9.110 | 3.366 | Up | 0.919 | DEFA4 |
| 100422913 | 0.010 | 1.873 | 7.549 | Up | 0.814 | MIR320E |
| 2210 | 5.980 | 16.183 | 1.436 | Up | 0.842 | FCGR1B |
| 57126 | 1.537 | 20.957 | 3.770 | Up | 0.951 | CD177 |
| 100423000 | 0.010 | 3.167 | 8.307 | Up | 0.888 | MIR3161 |
| 677814 | 0.367 | 3.110 | 3.084 | Up | 0.825 | SNORA31 |
| 11274 | 1.297 | 13.183 | 3.346 | Up | 0.932 | USP18 |
| 7001 | 30.110 | 69.547 | 1.208 | Up | 0.845 | PRDX2 |
| 27090 | 16.700 | 38.283 | 1.197 | Up | 0.837 | ST6GALNAC4 |
| 3148 | 15.073 | 37.613 | 1.319 | Up | 0.851 | HMGB2 |
| 64108 | 3.533 | 11.867 | 1.748 | Up | 0.861 | RTP4 |
| 6173 | 12.220 | 45.083 | 1.883 | Up | 0.895 | RPL36A |
| 3049 | 13.167 | 26.893 | 1.030 | Up | 0.805 | HBQ1 |
| 3512 | 54.400 | 129.500 | 1.251 | Up | 0.853 | IGJ |
| 669 | 27.617 | 199.080 | 2.850 | Up | 0.945 | BPGM |
| 1475 | 7.413 | 17.710 | 1.256 | Up | 0.828 | CSTA |
| 3017 | 7.927 | 18.530 | 1.225 | Up | 0.826 | HIST1H2BD |
| 219285 | 11.250 | 24.067 | 1.097 | Up | 0.815 | SAMD9L |
| 7052 | 2.243 | 10.027 | 2.160 | Up | 0.877 | TGM2 |
| 4938 | 21.977 | 83.097 | 1.919 | Up | 0.901 | OAS1 |
| 284422 | 13.627 | 38.647 | 1.504 | Up | 0.865 | C19orf77 |
| 3848 | 6.617 | 19.010 | 1.523 | Up | 0.854 | KRT1 |
| 51191 | 12.867 | 31.100 | 1.273 | Up | 0.842 | HERC5 |
| 439996 | 12.857 | 64.213 | 2.320 | Up | 0.924 | IFIT1B |
| 100134229 | 5.590 | 14.857 | 1.410 | Up | 0.836 | LOC100134229 |
| 1668 | 8.173 | 142.947 | 4.128 | Up | 0.972 | DEFA3 |
| 150000 | 5.893 | 16.870 | 1.517 | Up | 0.850 | ABCC13 |
| 388588 | 3.137 | 17.310 | 2.464 | Up | 0.910 | SMIM1 |
| 597 | 13.667 | 43.157 | 1.659 | Up | 0.885 | BCL2A1 |
| 51327 | 73.587 | 423.053 | 2.523 | Up | 0.933 | AHSP |
| 5909 | 2.167 | 21.193 | 3.290 | Up | 0.938 | RAP1GAP |
| 442918 | 0.010 | 3.933 | 8.620 | Up | 0.911 | MIR373 |
| 55363 | 10.253 | 58.297 | 2.507 | Up | 0.927 | HEMGN |
| 2996 | 0.493 | 4.797 | 3.281 | Up | 0.875 | GYPE |
| 6614 | 0.607 | 5.200 | 3.100 | Up | 0.876 | SIGLEC1 |
| 115362 | 36.543 | 75.427 | 1.045 | Up | 0.823 | GBP5 |
| 8804 | 23.570 | 64.750 | 1.458 | Up | 0.868 | CREG1 |
| 3045 | 48.277 | 296.887 | 2.621 | Up | 0.939 | HBD |
| 10561 | 9.293 | 65.213 | 2.811 | Up | 0.941 | IFI44 |
| 3781 | 2.483 | 7.597 | 1.613 | Up | 0.827 | KCNN2 |
| 4680 | 0.397 | 5.427 | 3.774 | Up | 0.899 | CEACAM6 |
| 100132417 | 6.377 | 30.983 | 2.281 | Up | 0.909 | FCGR1C |
| 8993 | 8.897 | 25.160 | 1.500 | Up | 0.858 | PGLYRP1 |
| 1991 | 0.660 | 9.207 | 3.802 | Up | 0.928 | ELANE |
| 100913187 | 0.883 | 5.113 | 2.533 | Up | 0.852 | APOBEC3A_B |
| 3665 | 10.297 | 28.030 | 1.445 | Up | 0.856 | IRF7 |
| 26499 | 4.923 | 12.330 | 1.324 | Up | 0.822 | PLEK2 |
| 441081 | 13.190 | 31.420 | 1.252 | Up | 0.840 | LOC441081 |
| 94240 | 10.563 | 47.940 | 2.182 | Up | 0.912 | EPSTI1 |
| 100423062 | 114.457 | 281.837 | 1.300 | Up | 0.861 | IGLL5 |
| 8807 | 11.857 | 24.987 | 1.075 | Up | 0.813 | IL18RAP |
| 8991 | 61.257 | 155.463 | 1.344 | Up | 0.864 | SELENBP1 |
| 100847035 | 1.687 | 7.667 | 2.184 | Up | 0.864 | MIR548AR |
| 4939 | 24.837 | 67.500 | 1.442 | Up | 0.866 | OAS2 |
| 2993 | 0.363 | 6.830 | 4.233 | Up | 0.923 | GYPA |
| 7504 | 2.867 | 18.850 | 2.717 | Up | 0.921 | XK |
| 1511 | 0.433 | 4.890 | 3.496 | Up | 0.885 | CTSG |
| 51218 | 114.203 | 270.427 | 1.244 | Up | 0.855 | GLRX5 |
| 4860 | 11.420 | 28.600 | 1.324 | Up | 0.847 | PNP |
| 759 | 36.913 | 648.157 | 4.134 | Up | 0.974 | CA1 |
| 3145 | 6.530 | 14.553 | 1.156 | Up | 0.807 | HMBS |
| 2633 | 28.787 | 83.827 | 1.542 | Up | 0.874 | GBP1 |
| 4318 | 10.400 | 22.920 | 1.140 | Up | 0.820 | MMP9 |
| 10900 | 9.980 | 29.230 | 1.550 | Up | 0.864 | RUNDC3A |
| 8780 | 23.413 | 50.187 | 1.100 | Up | 0.827 | RIOK3 |
| 389860 | 1.593 | 5.263 | 1.724 | Up | 0.806 | PAGE2B |
| 54855 | 13.740 | 47.220 | 1.781 | Up | 0.891 | FAM46C |
| 4353 | 0.437 | 4.077 | 3.223 | Up | 0.860 | MPO |
| 162466 | 111.867 | 230.623 | 1.044 | Up | 0.827 | PHOSPHO1 |
| 51237 | 4.983 | 17.613 | 1.821 | Up | 0.877 | MZB1 |
| 3050 | 3.930 | 13.300 | 1.759 | Up | 0.865 | HBZ |
| 114769 | 11.500 | 40.090 | 1.802 | Up | 0.890 | CARD16 |
| 6037 | 1.343 | 6.457 | 2.265 | Up | 0.855 | RNASE3 |
| 7130 | 7.690 | 21.147 | 1.459 | Up | 0.852 | TNFAIP6 |
| 24138 | 7.630 | 18.853 | 1.305 | Up | 0.835 | IFIT5 |
| 100616364 | 0.010 | 3.453 | 8.432 | Up | 0.898 | MIR4785 |
| 199675 | 5.850 | 27.217 | 2.218 | Up | 0.906 | C19orf59 |
| 2235 | 4.207 | 13.603 | 1.693 | Up | 0.863 | FECH |
| 3557 | 28.183 | 61.773 | 1.132 | Up | 0.833 | IL1RN |
| 4599 | 35.167 | 83.410 | 1.246 | Up | 0.851 | MX1 |
| 5359 | 11.707 | 30.347 | 1.374 | Up | 0.852 | PLSCR1 |
| 129607 | 7.620 | 33.633 | 2.142 | Up | 0.907 | CMPK2 |
| 6283 | 90.257 | 281.557 | 1.641 | Up | 0.894 | S100A12 |
| 6521 | 119.803 | 257.600 | 1.104 | Up | 0.837 | SLC4A1 |
| 56994 | 27.983 | 66.353 | 1.246 | Up | 0.849 | CHPT1 |
| 338339 | 4.330 | 12.643 | 1.546 | Up | 0.841 | CLEC4D |
| 10964 | 6.277 | 53.863 | 3.101 | Up | 0.945 | IFI44L |
| 6279 | 406.987 | 1828.610 | 2.168 | Up | 0.920 | S100A8 |
| 9976 | 12.063 | 26.550 | 1.138 | Up | 0.823 | CLEC2B |
| 3959 | 3.200 | 9.650 | 1.592 | Up | 0.841 | LGALS3BP |
| 2994 | 5.433 | 55.207 | 3.345 | Up | 0.952 | GYPB |
| 118932 | 1.077 | 4.950 | 2.201 | Up | 0.832 | ANKRD22 |
| 8519 | 363.373 | 887.267 | 1.288 | Up | 0.861 | IFITM1 |
| 11326 | 0.557 | 3.333 | 2.582 | Up | 0.816 | VSIG4 |
| 284486 | 0.687 | 7.330 | 3.416 | Up | 0.909 | THEM5 |
| 51513 | 1.527 | 5.637 | 1.884 | Up | 0.822 | ETV7 |
| 383 | 2.073 | 11.110 | 2.422 | Up | 0.895 | ARG1 |
| 3430 | 12.543 | 25.927 | 1.048 | Up | 0.808 | IFI35 |
| 100526831 | 0.010 | 1.900 | 7.570 | Up | 0.817 | SLX1B-SULT1A4 |
| 595100 | 0.010 | 3.170 | 8.308 | Up | 0.888 | SNORD18C |
| 26519 | 3.913 | 11.827 | 1.596 | Up | 0.851 | TIMM10 |
| 9381 | 0.163 | 5.427 | 5.054 | Up | 0.919 | OTOF |
| 3429 | 3.497 | 594.533 | 7.410 | Up | 0.994 | IFI27 |
| 4317 | 0.507 | 14.973 | 4.885 | Up | 0.960 | MMP8 |
| 25907 | 1.480 | 14.850 | 3.327 | Up | 0.935 | TMEM158 |
| 3437 | 71.477 | 292.107 | 2.031 | Up | 0.916 | IFIT3 |
| 100874300 | 1.957 | 5.883 | 1.588 | Up | 0.806 | SYNJ2-IT1 |
| 29126 | 2.327 | 8.240 | 1.824 | Up | 0.846 | CD274 |
| 8638 | 10.687 | 35.710 | 1.741 | Up | 0.887 | OASL |
| 339166 | 1.960 | 6.680 | 1.769 | Up | 0.829 | LOC339166 |
| 6535 | 16.180 | 34.477 | 1.091 | Up | 0.821 | SLC6A8 |
| 6563 | 8.547 | 19.733 | 1.207 | Up | 0.825 | SLC14A1 |
| 212 | 466.730 | 1476.987 | 1.662 | Up | 0.896 | ALAS2 |
| 4940 | 11.573 | 42.100 | 1.863 | Up | 0.893 | OAS3 |
| 23762 | 10.580 | 24.917 | 1.236 | Up | 0.835 | OSBP2 |
| 84418 | 16.930 | 33.983 | 1.005 | Up | 0.806 | CYSTM1 |
| 3240 | 1.993 | 10.783 | 2.436 | Up | 0.895 | HP |
| 1066 | 1.877 | 6.240 | 1.733 | Up | 0.821 | CES1 |
| 932 | 2.160 | 8.827 | 2.031 | Up | 0.867 | MS4A3 |
| 10124 | 1.300 | 5.353 | 2.042 | Up | 0.833 | ARL4A |
| 10661 | 7.723 | 22.670 | 1.553 | Up | 0.859 | KLF1 |
| 2209 | 5.683 | 21.497 | 1.919 | Up | 0.885 | FCGR1A |
| 6035 | 0.400 | 4.047 | 3.339 | Up | 0.864 | RNASE1 |
| 219855 | 8.697 | 19.170 | 1.140 | Up | 0.814 | SLC37A2 |
| 10321 | 0.623 | 6.913 | 3.471 | Up | 0.909 | CRISP3 |
| 5657 | 0.117 | 3.967 | 5.087 | Up | 0.895 | PRTN3 |
| 4061 | 43.070 | 256.557 | 2.575 | Up | 0.933 | LY6E |
| 2537 | 74.807 | 313.053 | 2.065 | Up | 0.917 | IFI6 |
| 54498 | 8.030 | 18.050 | 1.169 | Up | 0.817 | SMOX |
| 3934 | 6.260 | 84.457 | 3.754 | Up | 0.964 | LCN2 |
| 2766 | 51.893 | 110.210 | 1.087 | Up | 0.832 | GMPR |
| 26830 | 0.010 | 2.500 | 7.966 | Up | 0.859 | RNU5D-1 |
| 2003 | 17.697 | 72.157 | 2.028 | Up | 0.911 | ELK2AP |
| 116369 | 0.900 | 3.900 | 2.115 | Up | 0.805 | SLC26A8 |
| 692211 | 0.010 | 2.160 | 7.755 | Up | 0.838 | SNORD98 |
| 4057 | 2.743 | 32.690 | 3.575 | Up | 0.952 | LTF |
| 728358 | 26.867 | 524.370 | 4.287 | Up | 0.976 | DEFA1B |
| 820 | 4.373 | 27.553 | 2.655 | Up | 0.926 | CAMP |
| 55432 | 3.977 | 10.127 | 1.349 | Up | 0.814 | YOD1 |
| 9232 | 3.197 | 9.443 | 1.563 | Up | 0.828 | PTTG1 |
| 671 | 1.680 | 14.367 | 3.096 | Up | 0.926 | BPI |
| 6622 | 62.220 | 139.337 | 1.163 | Up | 0.843 | SNCA |
| 4502 | 21.277 | 71.977 | 1.758 | Up | 0.894 | MT2A |
| 6036 | 15.040 | 63.400 | 2.076 | Up | 0.912 | RNASE2 |
| 56729 | 3.193 | 14.297 | 2.163 | Up | 0.891 | RETN |
| 6478 | 24.823 | 76.427 | 1.622 | Up | 0.887 | SIAH2 |
| 219537 | 0.583 | 4.247 | 2.864 | Up | 0.852 | SMTNL1 |
| 116071 | 1.860 | 9.787 | 2.396 | Up | 0.890 | BATF2 |
| 8809 | 3.163 | 8.120 | 1.360 | Up | 0.801 | IL18R1 |
| 3434 | 13.817 | 66.123 | 2.259 | Up | 0.916 | IFIT1 |
| 760 | 6.477 | 17.400 | 1.426 | Up | 0.844 | CA2 |
| 306 | 8.553 | 29.143 | 1.769 | Up | 0.885 | ANXA3 |
| 1667 | 2.200 | 13.963 | 2.666 | Up | 0.913 | DEFA1 |
| 5610 | 12.413 | 25.133 | 1.018 | Up | 0.802 | EIF2AK2 |
| 100616211 | 0.010 | 3.777 | 8.561 | Up | 0.907 | MIR4709 |
| 100272216 | 16.253 | 38.830 | 1.256 | Up | 0.845 | LOC100272216 |
| 566 | 0.563 | 7.190 | 3.674 | Up | 0.914 | AZU1 |
| 10562 | 0.637 | 16.040 | 4.655 | Up | 0.959 | OLFM4 |
| 665 | 72.303 | 210.383 | 1.541 | Up | 0.878 | BNIP3L |
| 440288 | 12.470 | 36.303 | 1.542 | Up | 0.866 | LOC440288 |
| 10170 | 7.417 | 16.620 | 1.164 | Up | 0.812 | DHRS9 |
| 6280 | 2930.247 | 6855.203 | 1.226 | Up | 0.855 | S100A9 |
| 10410 | 405.797 | 1142.993 | 1.494 | Up | 0.877 | IFITM3 |
| 26010 | 2.060 | 6.793 | 1.721 | Up | 0.827 | SPATS2L |
| 3042 | 44.403 | 114.440 | 1.366 | Up | 0.864 | HBM |
| 9447 | 4.717 | 15.917 | 1.755 | Up | 0.871 | AIM2 |
| 9911 | 4.183 | 23.307 | 2.478 | Up | 0.916 | TMCC2 |
| 136319 | 0.010 | 8.210 | 9.681 | Up | 0.959 | MTPN |
| 55544 | 100.730 | 207.297 | 1.041 | Up | 0.826 | RBM38 |
| 6005 | 0.200 | 2.567 | 3.682 | Up | 0.820 | RHAG |
| 100873748 | 10.177 | 26.237 | 1.366 | Up | 0.849 | RNU6-33 |
| 3433 | 57.340 | 122.690 | 1.097 | Up | 0.834 | IFIT2 |
| 552900 | 0.010 | 1.807 | 7.497 | Up | 0.809 | BOLA2 |
| 514 | 76.183 | 214.070 | 1.491 | Up | 0.876 | ATP5E |
| 54739 | 35.430 | 90.237 | 1.349 | Up | 0.862 | XAF1 |
| 7264 | 22.767 | 51.460 | 1.177 | Up | 0.839 | TSTA3 |
| 100500849 | 1.830 | 5.640 | 1.624 | Up | 0.804 | MIR3916 |
| 100169764 | 3.653 | 0.283 | -3.689 | Down | 0.862 | RNA5S13 |
| 100169751 | 3.653 | 0.283 | -3.689 | Down | 0.862 | RNA5S1 |
| 57062 | 26.727 | 13.290 | -1.008 | Down | 0.801 | DDX24 |
| 11251 | 3.117 | 0.417 | -2.903 | Down | 0.819 | PTGDR2 |
| 8661 | 25.310 | 11.430 | -1.147 | Down | 0.823 | EIF3A |
| 1318 | 21.420 | 10.233 | -1.066 | Down | 0.806 | SLC31A2 |
| 3192 | 38.663 | 18.623 | -1.054 | Down | 0.815 | HNRNPU |
| 3823 | 6.300 | 1.833 | -1.781 | Down | 0.825 | KLRC3 |
| 2273 | 6.160 | 1.733 | -1.829 | Down | 0.826 | FHL1 |
| 4145 | 11.093 | 4.423 | -1.326 | Down | 0.817 | MATK |
| 8826 | 76.693 | 35.557 | -1.109 | Down | 0.832 | IQGAP1 |
| 84525 | 16.433 | 6.067 | -1.438 | Down | 0.843 | HOPX |
| 81539 | 14.963 | 6.300 | -1.248 | Down | 0.820 | SLC38A1 |
| 51744 | 14.323 | 5.183 | -1.466 | Down | 0.841 | CD244 |
| 100422970 | 15.313 | 2.953 | -2.374 | Down | 0.905 | MIR1273D |
| 11034 | 18.713 | 8.823 | -1.085 | Down | 0.804 | DSTN |
| 259197 | 7.510 | 2.347 | -1.678 | Down | 0.832 | NCR3 |
| 5730 | 8.173 | 1.967 | -2.055 | Down | 0.864 | PTGDS |
| 100169758 | 3.653 | 0.283 | -3.689 | Down | 0.862 | RNA5S7 |
| 9788 | 7.760 | 2.827 | -1.457 | Down | 0.807 | MTSS1 |
| 7535 | 24.963 | 11.807 | -1.080 | Down | 0.813 | ZAP70 |
| 3568 | 2.823 | 0.277 | -3.351 | Down | 0.824 | IL5RA |
| 5573 | 90.360 | 40.273 | -1.166 | Down | 0.843 | PRKAR1A |
| 130074 | 7.527 | 2.750 | -1.453 | Down | 0.804 | FAM168B |
| 283070 | 18.347 | 5.113 | -1.843 | Down | 0.879 | LOC283070 |
| 9249 | 5.630 | 1.767 | -1.672 | Down | 0.808 | DHRS3 |
| 1236 | 32.880 | 12.840 | -1.357 | Down | 0.852 | CCR7 |
| 100130231 | 29.347 | 12.143 | -1.273 | Down | 0.841 | LINC00861 |
| 921 | 12.990 | 5.443 | -1.255 | Down | 0.815 | CD5 |
| 53637 | 12.593 | 5.293 | -1.250 | Down | 0.813 | S1PR5 |
| 2534 | 36.917 | 17.647 | -1.065 | Down | 0.816 | FYN |
| 116496 | 58.507 | 28.223 | -1.052 | Down | 0.820 | FAM129A |
| 2316 | 66.303 | 32.773 | -1.017 | Down | 0.816 | FLNA |
| 406962 | 2.107 | 0.010 | -7.719 | Down | 0.834 | MIR186 |
| 100169755 | 3.653 | 0.283 | -3.689 | Down | 0.862 | RNA5S4 |
| 407025 | 8.367 | 2.910 | -1.524 | Down | 0.818 | MIR29B2 |
| 5551 | 64.030 | 21.137 | -1.599 | Down | 0.885 | PRF1 |
| 23344 | 19.357 | 6.397 | -1.597 | Down | 0.868 | ESYT1 |
| 2624 | 2.593 | 0.300 | -3.112 | Down | 0.802 | GATA2 |
| 9172 | 3.950 | 0.613 | -2.687 | Down | 0.837 | MYOM2 |
| 54541 | 11.703 | 4.620 | -1.341 | Down | 0.821 | DDIT4 |
| 3126 | 50.080 | 21.543 | -1.217 | Down | 0.844 | HLA-DRB4 |
| 51621 | 20.163 | 9.767 | -1.046 | Down | 0.800 | KLF13 |
| 100616387 | 32.357 | 13.163 | -1.298 | Down | 0.846 | MIR3064 |
| 6084 | 10.470 | 3.383 | -1.630 | Down | 0.848 | RNY1 |
| 1107 | 12.710 | 5.467 | -1.217 | Down | 0.810 | CHD3 |
| 113791 | 25.173 | 10.993 | -1.195 | Down | 0.830 | PIK3IP1 |
| 6907 | 14.287 | 6.010 | -1.249 | Down | 0.819 | TBL1X |
| 3575 | 81.530 | 25.133 | -1.698 | Down | 0.893 | IL7R |
| 6932 | 59.397 | 22.497 | -1.401 | Down | 0.862 | TCF7 |
| 223 | 15.560 | 6.580 | -1.242 | Down | 0.821 | ALDH9A1 |
| 100126351 | 2.993 | 0.010 | -8.226 | Down | 0.882 | MIR939 |
| 1178 | 37.867 | 17.083 | -1.148 | Down | 0.831 | CLC |
| 26986 | 401.213 | 197.230 | -1.024 | Down | 0.824 | PABPC1 |
| 27334 | 8.257 | 2.797 | -1.562 | Down | 0.819 | P2RY10 |
| 1656 | 28.800 | 13.020 | -1.145 | Down | 0.826 | DDX6 |
| 57091 | 9.943 | 3.643 | -1.448 | Down | 0.822 | CASS4 |
| 1901 | 21.687 | 10.093 | -1.103 | Down | 0.812 | S1PR1 |
| 10578 | 171.197 | 59.533 | -1.524 | Down | 0.877 | GNLY |
| 6480 | 23.330 | 10.717 | -1.122 | Down | 0.817 | ST6GAL1 |
| 5934 | 43.913 | 19.513 | -1.170 | Down | 0.836 | RBL2 |
| 56911 | 12.647 | 4.717 | -1.423 | Down | 0.832 | MAP3K7CL |
| 64781 | 14.043 | 6.247 | -1.169 | Down | 0.808 | CERK |
| 3423 | 43.207 | 20.447 | -1.079 | Down | 0.822 | IDS |
| 8773 | 32.983 | 16.433 | -1.005 | Down | 0.806 | SNAP23 |
| 11123 | 8.680 | 3.173 | -1.452 | Down | 0.814 | RCAN3 |
| 7048 | 45.247 | 22.487 | -1.009 | Down | 0.811 | TGFBR2 |
| 3716 | 61.347 | 23.553 | -1.381 | Down | 0.861 | JAK1 |
| 10594 | 16.500 | 6.433 | -1.359 | Down | 0.836 | PRPF8 |
| 221092 | 14.813 | 6.070 | -1.287 | Down | 0.825 | HNRNPUL2 |
| 170575 | 15.503 | 6.850 | -1.178 | Down | 0.813 | GIMAP1 |
| 7045 | 25.180 | 8.740 | -1.527 | Down | 0.860 | TGFBI |
| 2776 | 12.850 | 5.773 | -1.154 | Down | 0.800 | GNAQ |
| 767558 | 39.907 | 16.783 | -1.250 | Down | 0.844 | LUZP6 |
| 22821 | 19.517 | 8.583 | -1.185 | Down | 0.822 | RASA3 |
| 6601 | 14.193 | 6.360 | -1.158 | Down | 0.806 | SMARCC2 |
| 1436 | 14.713 | 4.907 | -1.584 | Down | 0.859 | CSF1R |
| 919 | 53.563 | 17.590 | -1.606 | Down | 0.884 | CD247 |
| 100422971 | 2.650 | 0.010 | -8.050 | Down | 0.867 | MIR4312 |
| 3394 | 16.567 | 6.637 | -1.320 | Down | 0.833 | IRF8 |
| 26822 | 2.470 | 0.010 | -7.948 | Down | 0.857 | SNORD14A |
| 51176 | 23.637 | 9.010 | -1.391 | Down | 0.849 | LEF1 |
| 84329 | 35.360 | 16.050 | -1.140 | Down | 0.828 | HVCN1 |
| 23547 | 3.373 | 0.583 | -2.532 | Down | 0.810 | LILRA4 |
| 22918 | 20.947 | 8.073 | -1.375 | Down | 0.845 | CD93 |
| 4627 | 83.810 | 31.637 | -1.406 | Down | 0.865 | MYH9 |
| 8553 | 12.617 | 4.637 | -1.444 | Down | 0.833 | BHLHE40 |
| 920 | 33.400 | 10.860 | -1.621 | Down | 0.879 | CD4 |
| 100169767 | 3.653 | 0.283 | -3.689 | Down | 0.862 | RNA5S16 |
| 89790 | 33.517 | 15.807 | -1.084 | Down | 0.818 | SIGLEC10 |
| 84658 | 34.907 | 13.280 | -1.394 | Down | 0.856 | EMR3 |
| 25777 | 44.347 | 19.627 | -1.176 | Down | 0.837 | SUN2 |
| 100132247 | 26.947 | 13.403 | -1.008 | Down | 0.802 | LOC100132247 |
| 3560 | 19.583 | 6.700 | -1.547 | Down | 0.856 | IL2RB |
| 3655 | 5.433 | 1.753 | -1.632 | Down | 0.802 | ITGA6 |
| 8148 | 31.767 | 12.880 | -1.302 | Down | 0.846 | TAF15 |
| 84187 | 19.140 | 7.183 | -1.414 | Down | 0.845 | TMEM164 |
| 3820 | 19.693 | 6.900 | -1.513 | Down | 0.854 | KLRB1 |
| 100873741 | 6.893 | 0.930 | -2.890 | Down | 0.890 | RNU6-8 |
| 4478 | 195.890 | 94.407 | -1.053 | Down | 0.828 | MSN |
| 7094 | 46.503 | 22.630 | -1.039 | Down | 0.816 | TLN1 |
| 821 | 35.693 | 17.637 | -1.017 | Down | 0.809 | CANX |
| 271 | 18.737 | 5.953 | -1.654 | Down | 0.871 | AMPD2 |
| 9302 | 3.567 | 0.010 | -8.478 | Down | 0.901 | SNORD26 |
| 2113 | 55.503 | 18.030 | -1.622 | Down | 0.885 | ETS1 |
| 10123 | 31.697 | 13.720 | -1.208 | Down | 0.835 | ARL4C |
| 100169768 | 3.653 | 0.283 | -3.689 | Down | 0.862 | RNA5S17 |
| 64919 | 7.370 | 2.370 | -1.637 | Down | 0.827 | BCL11B |
| 2162 | 39.030 | 19.290 | -1.017 | Down | 0.810 | F13A1 |
| 5583 | 27.417 | 11.453 | -1.259 | Down | 0.839 | PRKCH |
| 3118 | 11.553 | 4.593 | -1.331 | Down | 0.819 | HLA-DQA2 |
| 23524 | 26.093 | 12.083 | -1.111 | Down | 0.818 | SRRM2 |
| 26119 | 19.443 | 8.260 | -1.235 | Down | 0.828 | LDLRAP1 |
| 65108 | 21.467 | 9.950 | -1.109 | Down | 0.813 | MARCKSL1 |
| 123036 | 12.830 | 5.377 | -1.255 | Down | 0.814 | TC2N |
| 51673 | 5.563 | 1.217 | -2.193 | Down | 0.842 | TPPP3 |
| 6446 | 19.253 | 8.597 | -1.163 | Down | 0.817 | SGK1 |
| 9806 | 28.133 | 10.407 | -1.435 | Down | 0.855 | SPOCK2 |
| 85464 | 24.273 | 11.407 | -1.089 | Down | 0.814 | SSH2 |
| 100169753 | 3.653 | 0.283 | -3.689 | Down | 0.862 | RNA5S2 |
| 8436 | 17.873 | 7.717 | -1.212 | Down | 0.821 | SDPR |
| 677810 | 2.420 | 0.010 | -7.919 | Down | 0.854 | SNORA26 |
| 3125 | 46.370 | 21.637 | -1.100 | Down | 0.826 | HLA-DRB3 |
| 2208 | 6.050 | 2.010 | -1.590 | Down | 0.808 | FCER2 |
| 1108 | 17.103 | 7.680 | -1.155 | Down | 0.813 | CHD4 |
| 100169754 | 3.653 | 0.283 | -3.689 | Down | 0.862 | RNA5S3 |
| 100169763 | 3.653 | 0.283 | -3.689 | Down | 0.862 | RNA5S12 |
| 100287029 | 6.447 | 1.270 | -2.344 | Down | 0.865 | DDX11L10 |
| 924 | 31.657 | 12.743 | -1.313 | Down | 0.846 | CD7 |
| 100129083 | 13.020 | 4.570 | -1.510 | Down | 0.840 | LOC100129083 |
| 678 | 62.017 | 23.567 | -1.396 | Down | 0.863 | ZFP36L2 |
| 1975 | 73.123 | 30.647 | -1.255 | Down | 0.850 | EIF4B |
| 10949 | 12.057 | 5.193 | -1.215 | Down | 0.808 | HNRNPA0 |
| 23149 | 9.173 | 3.740 | -1.294 | Down | 0.800 | FCHO1 |
| 23193 | 17.487 | 7.953 | -1.137 | Down | 0.811 | GANAB |
| 129293 | 5.503 | 1.660 | -1.729 | Down | 0.810 | TRABD2A |
| 84433 | 9.867 | 3.973 | -1.312 | Down | 0.807 | CARD11 |
| 26999 | 23.717 | 10.523 | -1.172 | Down | 0.825 | CYFIP2 |
| 55313 | 50.743 | 23.783 | -1.093 | Down | 0.826 | CPPED1 |
| 619567 | 3.027 | 0.010 | -8.242 | Down | 0.883 | SNORD2 |
| 100616269 | 2.053 | 0.010 | -7.682 | Down | 0.829 | MIR4639 |
| 27250 | 35.550 | 16.937 | -1.070 | Down | 0.818 | PDCD4 |
| 2889 | 14.203 | 5.907 | -1.266 | Down | 0.821 | RAPGEF1 |
| 1292 | 3.703 | 0.823 | -2.169 | Down | 0.801 | COL6A2 |
| 1153 | 35.543 | 17.603 | -1.014 | Down | 0.807 | CIRBP |
| 2205 | 13.753 | 2.000 | -2.782 | Down | 0.914 | FCER1A |
| 100169766 | 3.653 | 0.283 | -3.689 | Down | 0.862 | RNA5S15 |
| 692157 | 2.140 | 0.010 | -7.741 | Down | 0.836 | SNORA16B |
| 27086 | 11.870 | 5.083 | -1.223 | Down | 0.807 | FOXP1 |
| 864 | 28.240 | 13.423 | -1.073 | Down | 0.813 | RUNX3 |
| 8761 | 19.777 | 7.507 | -1.398 | Down | 0.845 | PABPC4 |
| 6595 | 13.070 | 5.230 | -1.321 | Down | 0.824 | SMARCA2 |
| 51348 | 16.970 | 3.207 | -2.404 | Down | 0.908 | KLRF1 |
| 100169757 | 3.653 | 0.283 | -3.689 | Down | 0.862 | RNA5S6 |
| 641648 | 13.000 | 4.850 | -1.422 | Down | 0.833 | SNORD87 |
| 6653 | 45.983 | 20.893 | -1.138 | Down | 0.832 | SORL1 |
| 10125 | 10.280 | 4.363 | -1.236 | Down | 0.801 | RASGRP1 |
| 8445 | 5.843 | 1.713 | -1.770 | Down | 0.818 | DYRK2 |
| 3482 | 21.217 | 9.760 | -1.120 | Down | 0.814 | IGF2R |
| 4691 | 56.417 | 27.743 | -1.024 | Down | 0.815 | NCL |
| 5621 | 15.093 | 6.277 | -1.266 | Down | 0.823 | PRNP |
| 692196 | 2.310 | 0.010 | -7.852 | Down | 0.847 | SNORD76 |
| 3556 | 11.017 | 4.097 | -1.427 | Down | 0.826 | IL1RAP |
| 9214 | 43.637 | 18.633 | -1.228 | Down | 0.843 | FAIM3 |
| 476 | 29.643 | 12.140 | -1.288 | Down | 0.843 | ATP1A1 |
| 55022 | 3.007 | 0.373 | -3.010 | Down | 0.820 | PID1 |
| 4753 | 7.937 | 2.567 | -1.629 | Down | 0.832 | NELL2 |
| 196264 | 9.620 | 3.960 | -1.281 | Down | 0.802 | MPZL3 |
| 155038 | 14.310 | 6.517 | -1.135 | Down | 0.802 | GIMAP8 |
| 1521 | 53.170 | 20.327 | -1.387 | Down | 0.860 | CTSW |
| 50809 | 28.963 | 13.870 | -1.062 | Down | 0.812 | HP1BP3 |
| 1729 | 31.870 | 14.267 | -1.160 | Down | 0.829 | DIAPH1 |
| 9284 | 17.067 | 7.560 | -1.175 | Down | 0.815 | NPIP |
| 10768 | 11.797 | 5.220 | -1.176 | Down | 0.801 | AHCYL1 |
| 100169762 | 3.653 | 0.283 | -3.689 | Down | 0.862 | RNA5S11 |
| 81606 | 28.373 | 12.060 | -1.234 | Down | 0.837 | LBH |
| 4603 | 5.297 | 1.573 | -1.751 | Down | 0.808 | MYBL1 |
| 7090 | 28.713 | 13.993 | -1.037 | Down | 0.808 | TLE3 |
| 343413 | 11.750 | 4.273 | -1.459 | Down | 0.832 | FCRL6 |
| 11024 | 19.957 | 8.737 | -1.192 | Down | 0.823 | LILRA1 |
| 5175 | 61.217 | 28.097 | -1.124 | Down | 0.832 | PECAM1 |
| 117157 | 6.147 | 1.157 | -2.410 | Down | 0.863 | SH2D1B |
| 84852 | 13.727 | 4.973 | -1.465 | Down | 0.838 | ATP1A1OS |
| 6709 | 10.290 | 4.177 | -1.301 | Down | 0.808 | SPTAN1 |
| 5589 | 34.917 | 16.733 | -1.061 | Down | 0.816 | PRKCSH |
| 55690 | 30.223 | 12.730 | -1.247 | Down | 0.840 | PACS1 |
| 472 | 11.910 | 5.037 | -1.242 | Down | 0.809 | ATM |
| 6733 | 19.297 | 9.007 | -1.099 | Down | 0.808 | SRPK2 |
| 1880 | 15.380 | 5.403 | -1.509 | Down | 0.846 | GPR183 |
| 149345 | 5.827 | 1.113 | -2.388 | Down | 0.859 | SHISA4 |
| 100169765 | 3.653 | 0.283 | -3.689 | Down | 0.862 | RNA5S14 |
| 100302190 | 2.210 | 0.010 | -7.788 | Down | 0.841 | MIR1976 |
| 7294 | 11.290 | 3.407 | -1.729 | Down | 0.857 | TXK |
| 1606 | 19.413 | 9.310 | -1.060 | Down | 0.801 | DGKA |
| 100616358 | 3.763 | 0.150 | -4.649 | Down | 0.885 | MIR4800 |
| 100169756 | 3.653 | 0.283 | -3.689 | Down | 0.862 | RNA5S5 |
| 5266 | 83.077 | 35.797 | -1.215 | Down | 0.846 | PI3 |
| 100169761 | 3.653 | 0.283 | -3.689 | Down | 0.862 | RNA5S10 |
| 79971 | 11.240 | 4.220 | -1.413 | Down | 0.825 | WLS |
| 100169759 | 3.653 | 0.283 | -3.689 | Down | 0.862 | RNA5S8 |
| 10417 | 15.213 | 5.913 | -1.363 | Down | 0.833 | SPON2 |
| 57326 | 38.270 | 15.323 | -1.320 | Down | 0.851 | PBXIP1 |
| 80335 | 30.817 | 14.153 | -1.123 | Down | 0.823 | WDR82 |
| 3702 | 19.393 | 7.867 | -1.302 | Down | 0.835 | ITK |
| 6497 | 8.597 | 2.927 | -1.555 | Down | 0.822 | SKI |
| 246 | 4.220 | 0.897 | -2.235 | Down | 0.818 | ALOX15 |
| 9019 | 10.697 | 3.390 | -1.658 | Down | 0.851 | MPZL1 |
| 399665 | 11.523 | 4.160 | -1.470 | Down | 0.832 | FAM102A |
| 1028 | 4.790 | 0.917 | -2.386 | Down | 0.843 | CDKN1C |
| 3983 | 11.823 | 3.783 | -1.644 | Down | 0.854 | ABLIM1 |
| 81027 | 58.140 | 26.137 | -1.153 | Down | 0.836 | TUBB1 |
| 100616209 | 538.007 | 157.987 | -1.768 | Down | 0.900 | MIR4461 |
| 9289 | 12.313 | 5.227 | -1.236 | Down | 0.811 | GPR56 |
| 322 | 6.253 | 1.880 | -1.734 | Down | 0.821 | APBB1 |
| 6793 | 40.433 | 20.047 | -1.012 | Down | 0.809 | STK10 |
| 219771 | 17.780 | 8.463 | -1.071 | Down | 0.800 | CCNY |
| 79026 | 20.867 | 7.313 | -1.513 | Down | 0.855 | AHNAK |
| 51386 | 67.337 | 31.430 | -1.099 | Down | 0.830 | EIF3L |
| 619570 | 2.590 | 0.010 | -8.017 | Down | 0.864 | SNORD95 |
| 10801 | 64.427 | 28.757 | -1.164 | Down | 0.838 | 9-Sep |
| 27244 | 7.480 | 2.487 | -1.589 | Down | 0.825 | SESN1 |
| 6651 | 26.343 | 12.547 | -1.070 | Down | 0.812 | SON |
| 647979 | 23.977 | 11.167 | -1.102 | Down | 0.815 | LINC00657 |
| 23180 | 8.180 | 2.990 | -1.452 | Down | 0.810 | RFTN1 |
| 959 | 9.273 | 3.223 | -1.525 | Down | 0.824 | CD40LG |
| 407018 | 4.080 | 0.777 | -2.393 | Down | 0.827 | MIR27A |
| 7155 | 19.780 | 8.980 | -1.139 | Down | 0.815 | TOP2B |
| 1232 | 14.040 | 3.117 | -2.171 | Down | 0.891 | CCR3 |
| 1731 | 23.493 | 11.437 | -1.039 | Down | 0.803 | 1-Sep |
| 22838 | 18.400 | 8.087 | -1.186 | Down | 0.820 | RNF44 |

SLE, systemic lupus erythematosus; HC, healthy controls; FPKM, fragments per kilobase of exon model per million mapped reads.
