# Supplementary material for: Coagulation cascade and complement system in systemic lupus erythematosus
Source: Oncotarget. 2017 Dec 11;9(19):14862–81. doi: 10.18632/oncotarget.23206 (PMC5871083; doi:10.18632/oncotarget.23206)
Supplement: Supplementary file 3 [file oncotarget-09-14862-s003.docx]

**Supplementary Table 2. Selected GO biological processes enrichments identified among differentially expressed genes in SLE patients.**

| **Biological processes** | **Cluster frequency** | **Genome frequency of use** | ***p*-value** |
| --- | --- | --- | --- |
| [immune system process](http://amigo.geneontology.org/amigo/term/GO:0002376) | 83 out of 307 genes, 27.0% | 1106 out of 14596 genes, 7.6% | 0.000 |
| [defense response](http://amigo.geneontology.org/amigo/term/GO:0006952) | 50 out of 307 genes, 16.3% | 601 out of 14596 genes, 4.1% | 0.000 |
| [cytokine-mediated signaling pathway](http://amigo.geneontology.org/amigo/term/GO:0019221) | 23 out of 307 genes, 7.5% | 120 out of 14596 genes, 0.8% | 0.000 |
| [response to biotic stimulus](http://amigo.geneontology.org/amigo/term/GO:0009607) | 30 out of 307 genes, 9.8% | 360 out of 14596 genes, 2.5% | 0.000 |
| [response to stress](http://amigo.geneontology.org/amigo/term/GO:0006950) | 73 out of 307 genes, 23.8% | 1660 out of 14596 genes, 11.4% | 0.000 |
| [response to other organism](http://amigo.geneontology.org/amigo/term/GO:0051707) | 28 out of 307 genes, 9.1% | 337 out of 14596 genes, 2.3% | 0.000 |
| [response to stimulus](http://amigo.geneontology.org/amigo/term/GO:0050896) | 118 out of 307 genes, 38.4% | 3380 out of 14596 genes, 23.2% | 0.000 |
| [regulation of immune system process](http://amigo.geneontology.org/amigo/term/GO:0002682) | 37 out of 307 genes, 12.1% | 617 out of 14596 genes, 4.2% | 0.000 |
| [cell activation](http://amigo.geneontology.org/amigo/term/GO:0001775) | 32 out of 307 genes, 10.4% | 512 out of 14596 genes, 3.5% | 0.000 |
| [multi-organism process](http://amigo.geneontology.org/amigo/term/GO:0051704) | 37 out of 307 genes, 12.1% | 676 out of 14596 genes, 4.6% | 0.000 |
| [leukocyte activation](http://amigo.geneontology.org/amigo/term/GO:0045321) | 22 out of 307 genes, 7.2% | 289 out of 14596 genes, 2.0% | 0.000 |
| [cell killing](http://amigo.geneontology.org/amigo/term/GO:0001906) | 8 out of 307 genes, 2.6% | 33 out of 14596 genes, 0.2% | 0.000 |
| [endocytosis](http://amigo.geneontology.org/amigo/term/GO:0006897) | 14 out of 307 genes, 4.6% | 132 out of 14596 genes, 0.9% | 0.001 |
| [membrane invagination](http://amigo.geneontology.org/amigo/term/GO:0010324) | 14 out of 307 genes, 4.6% | 132 out of 14596 genes, 0.9% | 0.001 |
| [regulation of multi-organism process](http://amigo.geneontology.org/amigo/term/GO:0043900) | 10 out of 307 genes, 3.3% | 67 out of 14596 genes, 0.5% | 0.001 |
| [regulation of leukocyte activation](http://amigo.geneontology.org/amigo/term/GO:0002694) | 18 out of 307 genes, 5.9% | 230 out of 14596 genes, 1.6% | 0.002 |
| [gas transport](http://amigo.geneontology.org/amigo/term/GO:0015669) | 6 out of 307 genes, 2.0% | 21 out of 14596 genes, 0.1% | 0.003 |
| [regulation of cell activation](http://amigo.geneontology.org/amigo/term/GO:0050865) | 18 out of 307 genes, 5.9% | 242 out of 14596 genes, 1.7% | 0.003 |
| [positive regulation of leukocyte activation](http://amigo.geneontology.org/amigo/term/GO:0002696) | 13 out of 307 genes, 4.2% | 134 out of 14596 genes, 0.9% | 0.004 |
| [positive regulation of cell activation](http://amigo.geneontology.org/amigo/term/GO:0050867) | 13 out of 307 genes, 4.2% | 134 out of 14596 genes, 0.9% | 0.004 |
| [positive regulation of lymphocyte activation](http://amigo.geneontology.org/amigo/term/GO:0051251) | 12 out of 307 genes, 3.9% | 117 out of 14596 genes, 0.8% | 0.005 |
| [immune effector process](http://amigo.geneontology.org/amigo/term/GO:0002252) | 15 out of 307 genes, 4.9% | 181 out of 14596 genes, 1.2% | 0.006 |
| [immune system development](http://amigo.geneontology.org/amigo/term/GO:0002520) | 20 out of 307 genes, 6.5% | 308 out of 14596 genes, 2.1% | 0.007 |
| [hemopoiesis](http://amigo.geneontology.org/amigo/term/GO:0030097) | 16 out of 307 genes, 5.2% | 213 out of 14596 genes, 1.5% | 0.010 |
| [regulation of growth of symbiont in host](http://amigo.geneontology.org/amigo/term/GO:0044126) | 5 out of 307 genes, 1.6% | 16 out of 14596 genes, 0.1% | 0.012 |
| [lymphocyte activation](http://amigo.geneontology.org/amigo/term/GO:0046649) | 17 out of 307 genes, 5.5% | 243 out of 14596 genes, 1.7% | 0.013 |
| [lymphocyte costimulation](http://amigo.geneontology.org/amigo/term/GO:0031294) | 9 out of 307 genes, 2.9% | 71 out of 14596 genes, 0.5% | 0.014 |
| [regulation of symbiosis, encompassing mutualism through parasitism](http://amigo.geneontology.org/amigo/term/GO:0043903) | 5 out of 307 genes, 1.6% | 17 out of 14596 genes, 0.1% | 0.017 |
| [regulation of lymphocyte activation](http://amigo.geneontology.org/amigo/term/GO:0051249) | 15 out of 307 genes, 4.9% | 203 out of 14596 genes, 1.4% | 0.022 |
| [positive regulation of immune system process](http://amigo.geneontology.org/amigo/term/GO:0002684) | 21 out of 307 genes, 6.8% | 361 out of 14596 genes, 2.5% | 0.023 |
| [biological regulation](http://amigo.geneontology.org/amigo/term/GO:0065007) | 160 out of 307 genes, 52.1% | 5940 out of 14596 genes, 40.7% | 0.025 |
| [hemopoietic or lymphoid organ development](http://amigo.geneontology.org/amigo/term/GO:0048534) | 18 out of 307 genes, 5.9% | 283 out of 14596 genes, 1.9% | 0.027 |
| [cellular membrane organization](http://amigo.geneontology.org/amigo/term/GO:0016044) | 17 out of 307 genes, 5.5% | 258 out of 14596 genes, 1.8% | 0.028 |
| [membrane organization](http://amigo.geneontology.org/amigo/term/GO:0061024) | 17 out of 307 genes, 5.5% | 258 out of 14596 genes, 1.8% | 0.028 |
| [disruption by host of symbiont cells](http://amigo.geneontology.org/amigo/term/GO:0051852) | 3 out of 307 genes, 1.0% | 4 out of 14596 genes, 0.0% | 0.031 |
| [cell surface receptor linked signaling pathway](http://amigo.geneontology.org/amigo/term/GO:0007166) | 65 out of 307 genes, 21.2% | 1894 out of 14596 genes, 13.0% | 0.031 |
| [response to cytokine stimulus](http://amigo.geneontology.org/amigo/term/GO:0034097) | 9 out of 307 genes, 2.9% | 81 out of 14596 genes, 0.6% | 0.042 |
| [modification by host of symbiont morphology or physiology](http://amigo.geneontology.org/amigo/term/GO:0051851) | 4 out of 307 genes, 1.3% | 12 out of 14596 genes, 0.1% | 0.071 |
| [leukocyte differentiation](http://amigo.geneontology.org/amigo/term/GO:0002521) | 12 out of 307 genes, 3.9% | 154 out of 14596 genes, 1.1% | 0.086 |

GO, gene ontology; SLE, systemic lupus erythematosus.
