# Supplementary material for: Coagulation cascade and complement system in systemic lupus erythematosus
Source: Oncotarget. 2017 Dec 11;9(19):14862–81. doi: 10.18632/oncotarget.23206 (PMC5871083; doi:10.18632/oncotarget.23206)
Supplement: Supplementary file 4 [file oncotarget-09-14862-s004.docx]

**Supplementary Table 4. Identification of proteins that are differentially displayed in SLE patients using iTRAQ.**

| **Accession** | **Fold change T1** | **Fold change T2** | **Fold change T3** | **Fold change mean** | **Up/**  **Down** |
| --- | --- | --- | --- | --- | --- |
| sp\|P35908\|K22E_HUMAN | 3.492 | 3.213 | 3.464 | 3.390 | Up |
| sp\|P23142\|FBLN1_HUMAN | 1.29 | 1.319 | 1.348 | 1.319 | Up |
| sp\|P18428\|LBP_HUMAN | 2.321 | 2.372 | 2.295 | 2.329 | Up |
| sp\|P09960\|LKHA4_HUMAN | 1.683 | 1.865 | 1.757 | 1.768 | Up |
| sp\|P03950\|ANGI_HUMAN | 1.585 | 1.624 | 1.675 | 1.628 | Up |
| sp\|P01608\|KV116_HUMAN | 1.35 | 1.217 | 1.283 | 1.283 | Up |
| sp\|P23381\|SYWC_HUMAN | 2.619 | 3.337 | 2.663 | 2.873 | Up |
| tr\|G3V2W1\|G3V2W1_HUMAN | 1.335 | 1.398 | 1.365 | 1.366 | Up |
| sp\|P19652\|A1AG2_HUMAN | 1.549 | 1.388 | 1.419 | 1.452 | Up |
| sp\|O76076\|WISP2_HUMAN | 1.677 | 1.635 | 1.675 | 1.662 | Up |
| sp\|P05814\|CASB_HUMAN | 2.86 | 3.153 | 3.51 | 3.174 | Up |
| tr\|H0Y7V6\|H0Y7V6_HUMAN | 2.051 | 1.939 | 2.403 | 2.131 | Up |
| sp\|P34096\|RNAS4_HUMAN | 1.579 | 1.583 | 1.634 | 1.599 | Up |
| sp\|Q86UD1\|OAF_HUMAN | 1.317 | 1.368 | 1.236 | 1.307 | Up |
| sp\|P22891\|PROZ_HUMAN | 1.688 | 1.385 | 1.584 | 1.552 | Up |
| sp\|P15291\|B4GT1_HUMAN | 2.473 | 2.23 | 2.611 | 2.438 | Up |
| tr\|H0Y512\|H0Y512_HUMAN | 1.57 | 1.627 | 1.576 | 1.591 | Up |
| sp\|P04264\|K2C1_HUMAN | 1.795 | 1.866 | 1.797 | 1.819 | Up |
| tr\|G3V4U0\|G3V4U0_HUMAN | 1.769 | 2.034 | 1.951 | 1.918 | Up |
| sp\|P08294\|SODE_HUMAN | 1.471 | 1.446 | 1.392 | 1.436 | Up |
| sp\|Q92743\|HTRA1_HUMAN | 2.055 | 1.901 | 1.982 | 1.979 | Up |
| tr\|G3V0E5\|G3V0E5_HUMAN | 1.399 | 1.455 | 1.404 | 1.419 | Up |
| sp\|P07900\|HS90A_HUMAN | 1.671 | 1.754 | 1.901 | 1.775 | Up |
| sp\|P08571\|CD14_HUMAN | 2.196 | 2.01 | 2.011 | 2.072 | Up |
| tr\|F5H8B0\|F5H8B0_HUMAN | 1.407 | 1.631 | 1.481 | 1.506 | Up |
| sp\|P10645\|CMGA_HUMAN | 2.059 | 2.01 | 1.95 | 2.006 | Up |
| sp\|P22692\|IBP4_HUMAN | 1.486 | 1.521 | 1.648 | 1.552 | Up |
| sp\|P01619\|KV301_HUMAN | 1.4 | 1.642 | 1.655 | 1.566 | Up |
| sp\|P14543\|NID1_HUMAN | 1.458 | 1.414 | 1.444 | 1.439 | Up |
| sp\|P55058\|PLTP_HUMAN | 1.314 | 1.301 | 1.229 | 1.281 | Up |
| sp\|Q8WWA0\|ITLN1_HUMAN | 1.262 | 1.336 | 1.355 | 1.318 | Up |
| sp\|Q14515\|SPRL1_HUMAN | 1.531 | 1.449 | 1.373 | 1.451 | Up |
| sp\|P27797\|CALR_HUMAN | 1.729 | 1.757 | 1.572 | 1.686 | Up |
| sp\|P08254\|MMP3_HUMAN | 3.075 | 2.839 | 3.055 | 2.990 | Up |
| sp\|Q8NI99\|ANGL6_HUMAN | 1.231 | 1.376 | 1.299 | 1.302 | Up |
| sp\|P32119\|PRDX2_HUMAN | 1.215 | 1.183 | 1.202 | 1.200 | Up |
| sp\|Q9UNW1\|MINP1_HUMAN | 1.374 | 1.444 | 1.436 | 1.418 | Up |
| sp\|P02763\|A1AG1_HUMAN | 2.564 | 2.528 | 2.413 | 2.502 | Up |
| sp\|P01011\|AACT_HUMAN | 1.82 | 1.744 | 1.844 | 1.803 | Up |
| tr\|B1ALD9\|B1ALD9_HUMAN | 1.65 | 1.647 | 1.492 | 1.596 | Up |
| sp\|Q92954\|PRG4_HUMAN | 1.506 | 1.548 | 1.539 | 1.531 | Up |
| tr\|E7ENL6\|E7ENL6_HUMAN | 1.833 | 1.736 | 1.676 | 1.748 | Up |
| sp\|O00187\|MASP2_HUMAN | 1.378 | 1.357 | 1.333 | 1.356 | Up |
| sp\|O95428\|PPN_HUMAN | 1.732 | 1.678 | 1.961 | 1.790 | Up |
| tr\|A0A075B6K4\|A0A075B6K4_HUMAN | 2.486 | 2.572 | 2.787 | 2.615 | Up |
| sp\|P01714\|LV301_HUMAN | 1.697 | 2.045 | 1.796 | 1.846 | Up |
| sp\|P05109\|S10A8_HUMAN | 1.741 | 1.674 | 1.597 | 1.671 | Up |
| tr\|A0A087WZW8\|A0A087WZW8_HUMAN | 2.101 | 2.281 | 1.702 | 2.028 | Up |
| sp\|P23083\|HV103_HUMAN | 1.532 | 1.505 | 1.689 | 1.575 | Up |
| sp\|P04275\|VWF_HUMAN | 1.556 | 1.55 | 1.539 | 1.548 | Up |
| sp\|P00738\|HPT_HUMAN | 1.502 | 1.566 | 1.677 | 1.582 | Up |
| tr\|A0A087WTM7\|A0A087WTM7_HUMAN | 1.312 | 1.395 | 1.27 | 1.326 | Up |
| sp\|P01625\|KV402_HUMAN | 1.701 | 1.814 | 1.63 | 1.715 | Up |
| sp\|Q08830\|FGL1_HUMAN | 3.057 | 3.198 | 2.614 | 2.956 | Up |
| tr\|E7EUT5\|E7EUT5_HUMAN | 1.23 | 1.373 | 1.31 | 1.304 | Up |
| sp\|Q9Y287\|ITM2B_HUMAN | 1.677 | 1.759 | 1.636 | 1.691 | Up |
| sp\|Q12805\|FBLN3_HUMAN | 1.62 | 1.561 | 1.604 | 1.595 | Up |
| tr\|H7BXV5\|H7BXV5_HUMAN | 1.289 | 1.284 | 1.313 | 1.295 | Up |
| sp\|P35527\|K1C9_HUMAN | 1.555 | 1.315 | 1.308 | 1.393 | Up |
| tr\|A0A0B4J1V5\|A0A0B4J1V5_HUMAN | 6.682 | 6.749 | 6.182 | 6.538 | Up |
| tr\|A0A087WYC5\|A0A087WYC5_HUMAN | 2.943 | 2.945 | 2.544 | 2.811 | Up |
| sp\|P13645\|K1C10_HUMAN | 1.832 | 1.894 | 1.832 | 1.853 | Up |
| sp\|P01717\|LV403_HUMAN | 2.168 | 2.23 | 2.339 | 2.246 | Up |
| tr\|C9J8S2\|C9J8S2_HUMAN | 1.268 | 1.254 | 1.228 | 1.250 | Up |
| tr\|A0A0B4J2B5\|A0A0B4J2B5_HUMAN | 1.157 | 1.458 | 1.407 | 1.341 | Up |
| sp\|O00391\|QSOX1_HUMAN | 1.367 | 1.342 | 1.337 | 1.349 | Up |
| tr\|A0A0B4J1X8\|A0A0B4J1X8_HUMAN | 1.744 | 1.715 | 1.79 | 1.750 | Up |
| sp\|P01768\|HV307_HUMAN | 1.538 | 1.551 | 1.582 | 1.557 | Up |
| tr\|C9J6H2\|C9J6H2_HUMAN | 1.529 | 1.68 | 1.589 | 1.599 | Up |
| sp\|P30041\|PRDX6_HUMAN | 1.342 | 1.311 | 1.221 | 1.291 | Up |
| tr\|C9JEV0\|C9JEV0_HUMAN | 1.454 | 1.618 | 1.481 | 1.518 | Up |
| sp\|P10643\|CO7_HUMAN | 1.272 | 1.249 | 1.311 | 1.277 | Up |
| sp\|P00451\|FA8_HUMAN | 1.592 | 1.601 | 1.633 | 1.609 | Up |
| tr\|A0A024R3W6\|A0A024R3W6_HUMAN | 1.454 | 1.53 | 1.466 | 1.483 | Up |
| sp\|Q8NBP7\|PCSK9_HUMAN | 1.625 | 1.633 | 1.655 | 1.638 | Up |
| sp\|P18065\|IBP2_HUMAN | 1.711 | 1.733 | 1.671 | 1.705 | Up |
| tr\|A0A087X0N5\|A0A087X0N5_HUMAN | 1.608 | 1.62 | 1.569 | 1.599 | Up |
| sp\|P08519\|APOA_HUMAN | 1.609 | 1.642 | 1.586 | 1.612 | Up |
| sp\|P61626\|LYSC_HUMAN | 1.301 | 1.362 | 1.342 | 1.335 | Up |
| sp\|P00709\|LALBA_HUMAN | 5.727 | 6.842 | 6.684 | 6.418 | Up |
| sp\|Q06033\|ITIH3_HUMAN | 1.646 | 1.586 | 1.606 | 1.613 | Up |
| sp\|P00740\|FA9_HUMAN | 1.466 | 1.38 | 1.441 | 1.429 | Up |
| sp\|P05543\|THBG_HUMAN | 1.28 | 1.325 | 1.333 | 1.313 | Up |
| tr\|I3L145\|I3L145_HUMAN | 1.431 | 1.364 | 1.385 | 1.393 | Up |
| sp\|P55056\|APOC4_HUMAN | 1.325 | 1.279 | 1.319 | 1.308 | Up |
| sp\|Q15582\|BGH3_HUMAN | 1.411 | 1.194 | 1.246 | 1.284 | Up |
| sp\|P04217\|A1BG_HUMAN | 1.288 | 1.414 | 1.409 | 1.370 | Up |
| sp\|P51884\|LUM_HUMAN | 0.848 | 0.815 | 0.802 | 0.822 | Down |
| sp\|P04180\|LCAT_HUMAN | 0.764 | 0.824 | 0.82 | 0.803 | Down |
| sp\|P20851\|C4BPB_HUMAN | 0.663 | 0.694 | 0.631 | 0.663 | Down |
| tr\|D6REX5\|D6REX5_HUMAN | 0.551 | 0.658 | 0.543 | 0.584 | Down |
| tr\|H9KV48\|H9KV48_HUMAN | 0.729 | 0.676 | 0.694 | 0.700 | Down |
| sp\|P00748\|FA12_HUMAN | 0.804 | 0.853 | 0.831 | 0.829 | Down |
| sp\|P02776\|PLF4_HUMAN | 0.528 | 0.556 | 0.547 | 0.544 | Down |
| sp\|P06727\|APOA4_HUMAN | 0.699 | 0.707 | 0.676 | 0.694 | Down |
| tr\|D6RAR4\|D6RAR4_HUMAN | 0.746 | 0.734 | 0.79 | 0.757 | Down |
| tr\|A0A087WTE1\|A0A087WTE1_HUMAN | 0.699 | 0.691 | 0.742 | 0.711 | Down |
| sp\|P02760\|AMBP_HUMAN | 0.805 | 0.82 | 0.857 | 0.827 | Down |
| sp\|P01591\|IGJ_HUMAN | 0.673 | 0.76 | 0.726 | 0.720 | Down |
| sp\|P01008\|ANT3_HUMAN | 0.576 | 0.628 | 0.61 | 0.605 | Down |
| tr\|C9JV77\|C9JV77_HUMAN | 0.689 | 0.662 | 0.673 | 0.675 | Down |
| tr\|A0A075B6H6\|A0A075B6H6_HUMAN | 0.743 | 0.748 | 0.757 | 0.749 | Down |
| tr\|E7EQB2\|E7EQB2_HUMAN | 0.816 | 0.762 | 0.737 | 0.772 | Down |
| sp\|P0C0L4\|CO4A_HUMAN | 0.577 | 0.786 | 0.795 | 0.719 | Down |
| sp\|P02743\|SAMP_HUMAN | 0.778 | 0.814 | 0.868 | 0.820 | Down |
| sp\|P07225\|PROS_HUMAN | 0.801 | 0.822 | 0.798 | 0.807 | Down |
| sp\|P04196\|HRG_HUMAN | 0.721 | 0.741 | 0.757 | 0.740 | Down |
| sp\|P00488\|F13A_HUMAN | 0.632 | 0.651 | 0.665 | 0.649 | Down |
| tr\|A0A075B6R9\|A0A075B6R9_HUMAN | 0.638 | 0.664 | 0.73 | 0.677 | Down |
| tr\|A0A0A0MSV6\|A0A0A0MSV6_HUMAN | 0.703 | 0.716 | 0.662 | 0.694 | Down |
| sp\|P02775\|CXCL7_HUMAN | 0.509 | 0.561 | 0.465 | 0.512 | Down |

SLE, systemic lupus erythematosus; iTRAQ, isobaric tag for relative and absolute quantitation; T, technical repeat.
