# Supplementary material for: Coagulation cascade and complement system in systemic lupus erythematosus
Source: Oncotarget. 2017 Dec 11;9(19):14862–81. doi: 10.18632/oncotarget.23206 (PMC5871083; doi:10.18632/oncotarget.23206)
Supplement: Supplementary file 5 [file oncotarget-09-14862-s005.docx]

**Supplementary Table 5. Selected GO biological processes enrichments identified among differentially expressed proteins in SLE patients.**

| **biological processes** | **Cluster frequency** | **Protein frequency of use** | ***p*-value** |
| --- | --- | --- | --- |
| [extracellular matrix organization](http://amigo.geneontology.org/amigo/term/GO:0030198) | 11 out of 95 genes, 11.6% | 24 out of 490 genes, 4.9% | 0.002 |
| [extracellular structure organization](http://amigo.geneontology.org/amigo/term/GO:0043062) | 11 out of 95 genes, 11.6% | 24 out of 490 genes, 4.9% | 0.002 |
| [inflammatory response](http://amigo.geneontology.org/amigo/term/GO:0006954) | 15 out of 95 genes, 15.8% | 41 out of 490 genes, 8.4% | 0.005 |
| [visual perception](http://amigo.geneontology.org/amigo/term/GO:0007601) | 4 out of 95 genes, 4.2% | 5 out of 490 genes, 1.0% | 0.006 |
| [positive regulation of tissue remodeling](http://amigo.geneontology.org/amigo/term/GO:0034105) | 4 out of 95 genes, 4.2% | 5 out of 490 genes, 1.0% | 0.006 |
| [sensory perception of light stimulus](http://amigo.geneontology.org/amigo/term/GO:0050953) | 4 out of 95 genes, 4.2% | 5 out of 490 genes, 1.0% | 0.006 |
| [acute inflammatory response](http://amigo.geneontology.org/amigo/term/GO:0002526) | 10 out of 95 genes, 10.5% | 24 out of 490 genes, 4.9% | 0.008 |
| [response to stimulus](http://amigo.geneontology.org/amigo/term/GO:0050896) | 71 out of 95 genes, 74.7% | 321 out of 490 genes, 65.5% | 0.022 |
| [response to superoxide](http://amigo.geneontology.org/amigo/term/GO:0000303) | 3 out of 95 genes, 3.2% | 4 out of 490 genes, 0.8% | 0.024 |
| [superoxide metabolic process](http://amigo.geneontology.org/amigo/term/GO:0006801) | 3 out of 95 genes, 3.2% | 4 out of 490 genes, 0.8% | 0.024 |
| [post-embryonic development](http://amigo.geneontology.org/amigo/term/GO:0009791) | 3 out of 95 genes, 3.2% | 4 out of 490 genes, 0.8% | 0.024 |
| [removal of superoxide radicals](http://amigo.geneontology.org/amigo/term/GO:0019430) | 3 out of 95 genes, 3.2% | 4 out of 490 genes, 0.8% | 0.024 |
| [cellular response to superoxide](http://amigo.geneontology.org/amigo/term/GO:0071451) | 3 out of 95 genes, 3.2% | 4 out of 490 genes, 0.8% | 0.024 |
| [regulation of tissue remodeling](http://amigo.geneontology.org/amigo/term/GO:0034103) | 4 out of 95 genes, 4.2% | 7 out of 490 genes, 1.4% | 0.029 |
| [defense response](http://amigo.geneontology.org/amigo/term/GO:0006952) | 31 out of 95 genes, 32.6% | 122 out of 490 genes, 24.9% | 0.037 |
| [lactose metabolic process](http://amigo.geneontology.org/amigo/term/GO:0005988) | 2 out of 95 genes, 2.1% | 2 out of 490 genes, 0.4% | 0.037 |
| [lactose biosynthetic process](http://amigo.geneontology.org/amigo/term/GO:0005989) | 2 out of 95 genes, 2.1% | 2 out of 490 genes, 0.4% | 0.037 |
| [response to vitamin A](http://amigo.geneontology.org/amigo/term/GO:0033189) | 2 out of 95 genes, 2.1% | 2 out of 490 genes, 0.4% | 0.037 |
| [response to stilbenoid](http://amigo.geneontology.org/amigo/term/GO:0035634) | 2 out of 95 genes, 2.1% | 2 out of 490 genes, 0.4% | 0.037 |
| [fibrinolysis](http://amigo.geneontology.org/amigo/term/GO:0042730) | 2 out of 95 genes, 2.1% | 2 out of 490 genes, 0.4% | 0.037 |
| [regulation of acrosome reaction](http://amigo.geneontology.org/amigo/term/GO:0060046) | 2 out of 95 genes, 2.1% | 2 out of 490 genes, 0.4% | 0.037 |
| [response to lipoteichoic acid](http://amigo.geneontology.org/amigo/term/GO:0070391) | 2 out of 95 genes, 2.1% | 2 out of 490 genes, 0.4% | 0.037 |
| [cellular response to lipoteichoic acid](http://amigo.geneontology.org/amigo/term/GO:0071223) | 2 out of 95 genes, 2.1% | 2 out of 490 genes, 0.4% | 0.037 |
| [response to phenylpropanoid](http://amigo.geneontology.org/amigo/term/GO:0080184) | 2 out of 95 genes, 2.1% | 2 out of 490 genes, 0.4% | 0.037 |
| [regulation of cell growth](http://amigo.geneontology.org/amigo/term/GO:0001558) | 7 out of 95 genes, 7.4% | 18 out of 490 genes, 3.7% | 0.041 |
| [acute-phase response](http://amigo.geneontology.org/amigo/term/GO:0006953) | 7 out of 95 genes, 7.4% | 18 out of 490 genes, 3.7% | 0.041 |
| [response to wounding](http://amigo.geneontology.org/amigo/term/GO:0009611) | 31 out of 95 genes, 32.6% | 123 out of 490 genes, 25.1% | 0.042 |
| [cellular response to oxidative stress](http://amigo.geneontology.org/amigo/term/GO:0034599) | 5 out of 95 genes, 5.3% | 11 out of 490 genes, 2.2% | 0.043 |
| [cellular response to reactive oxygen species](http://amigo.geneontology.org/amigo/term/GO:0034614) | 5 out of 95 genes, 5.3% | 11 out of 490 genes, 2.2% | 0.043 |
| [digestion](http://amigo.geneontology.org/amigo/term/GO:0007586) | 3 out of 95 genes, 3.2% | 5 out of 490 genes, 1.0% | 0.052 |
| [positive regulation of tumor necrosis factor production](http://amigo.geneontology.org/amigo/term/GO:0032760) | 3 out of 95 genes, 3.2% | 5 out of 490 genes, 1.0% | 0.052 |
| [positive regulation of blood coagulation](http://amigo.geneontology.org/amigo/term/GO:0030194) | 5 out of 95 genes, 5.3% | 12 out of 490 genes, 2.4% | 0.062 |
| [defense response to bacterium](http://amigo.geneontology.org/amigo/term/GO:0042742) | 5 out of 95 genes, 5.3% | 12 out of 490 genes, 2.4% | 0.062 |
| [positive regulation of coagulation](http://amigo.geneontology.org/amigo/term/GO:0050820) | 5 out of 95 genes, 5.3% | 12 out of 490 genes, 2.4% | 0.062 |
| [positive regulation of hemostasis](http://amigo.geneontology.org/amigo/term/GO:1900048) | 5 out of 95 genes, 5.3% | 12 out of 490 genes, 2.4% | 0.062 |
| [response to bacterium](http://amigo.geneontology.org/amigo/term/GO:0009617) | 9 out of 95 genes, 9.5% | 28 out of 490 genes, 5.7% | 0.071 |
| [response to inorganic substance](http://amigo.geneontology.org/amigo/term/GO:0010035) | 13 out of 95 genes, 13.7% | 45 out of 490 genes, 9.2% | 0.072 |
| [peptidyl-glutamic acid carboxylation](http://amigo.geneontology.org/amigo/term/GO:0017187) | 4 out of 95 genes, 4.2% | 9 out of 490 genes, 1.8% | 0.076 |
| [peptidyl-glutamic acid modification](http://amigo.geneontology.org/amigo/term/GO:0018200) | 4 out of 95 genes, 4.2% | 9 out of 490 genes, 1.8% | 0.076 |
| [protein carboxylation](http://amigo.geneontology.org/amigo/term/GO:0018214) | 4 out of 95 genes, 4.2% | 9 out of 490 genes, 1.8% | 0.076 |
| [response to other organism](http://amigo.geneontology.org/amigo/term/GO:0051707) | 11 out of 95 genes, 11.6% | 37 out of 490 genes, 7.6% | 0.080 |
| [cellular response to oxygen-containing compound](http://amigo.geneontology.org/amigo/term/GO:1901701) | 11 out of 95 genes, 11.6% | 37 out of 490 genes, 7.6% | 0.080 |
| [multicellular organismal process](http://amigo.geneontology.org/amigo/term/GO:0032501) | 54 out of 95 genes, 56.8% | 245 out of 490 genes, 50.0% | 0.085 |
| [response to oxygen-containing compound](http://amigo.geneontology.org/amigo/term/GO:1901700) | 18 out of 95 genes, 18.9% | 69 out of 490 genes, 14.1% | 0.090 |
| [response to biotic stimulus](http://amigo.geneontology.org/amigo/term/GO:0009607) | 11 out of 95 genes, 11.6% | 38 out of 490 genes, 7.8% | 0.094 |
| [disaccharide metabolic process](http://amigo.geneontology.org/amigo/term/GO:0005984) | 2 out of 95 genes, 2.1% | 3 out of 490 genes, 0.6% | 0.098 |
| [Toll signaling pathway](http://amigo.geneontology.org/amigo/term/GO:0008063) | 2 out of 95 genes, 2.1% | 3 out of 490 genes, 0.6% | 0.098 |
| [oligosaccharide biosynthetic process](http://amigo.geneontology.org/amigo/term/GO:0009312) | 2 out of 95 genes, 2.1% | 3 out of 490 genes, 0.6% | 0.098 |
| [regulation of hydrogen peroxide metabolic process](http://amigo.geneontology.org/amigo/term/GO:0010310) | 2 out of 95 genes, 2.1% | 3 out of 490 genes, 0.6% | 0.098 |
| [protein autoprocessing](http://amigo.geneontology.org/amigo/term/GO:0016540) | 2 out of 95 genes, 2.1% | 3 out of 490 genes, 0.6% | 0.098 |
| [negative regulation of translation](http://amigo.geneontology.org/amigo/term/GO:0017148) | 2 out of 95 genes, 2.1% | 3 out of 490 genes, 0.6% | 0.098 |
| [regulation of exocytosis](http://amigo.geneontology.org/amigo/term/GO:0017157) | 2 out of 95 genes, 2.1% | 3 out of 490 genes, 0.6% | 0.098 |
| [regulation of calcium ion-dependent exocytosis](http://amigo.geneontology.org/amigo/term/GO:0017158) | 2 out of 95 genes, 2.1% | 3 out of 490 genes, 0.6% | 0.098 |
| [sperm motility](http://amigo.geneontology.org/amigo/term/GO:0030317) | 2 out of 95 genes, 2.1% | 3 out of 490 genes, 0.6% | 0.098 |
| [positive regulation of bone resorption](http://amigo.geneontology.org/amigo/term/GO:0045780) | 2 out of 95 genes, 2.1% | 3 out of 490 genes, 0.6% | 0.098 |
| [disaccharide biosynthetic process](http://amigo.geneontology.org/amigo/term/GO:0046351) | 2 out of 95 genes, 2.1% | 3 out of 490 genes, 0.6% | 0.098 |
| [response to copper ion](http://amigo.geneontology.org/amigo/term/GO:0046688) | 2 out of 95 genes, 2.1% | 3 out of 490 genes, 0.6% | 0.098 |
| [positive regulation of bone remodeling](http://amigo.geneontology.org/amigo/term/GO:0046852) | 2 out of 95 genes, 2.1% | 3 out of 490 genes, 0.6% | 0.098 |
| [chaperone-mediated protein complex assembly](http://amigo.geneontology.org/amigo/term/GO:0051131) | 2 out of 95 genes, 2.1% | 3 out of 490 genes, 0.6% | 0.098 |
| [regulation of cellular response to oxidative stress](http://amigo.geneontology.org/amigo/term/GO:1900407) | 2 out of 95 genes, 2.1% | 3 out of 490 genes, 0.6% | 0.098 |
| [regulation of response to reactive oxygen species](http://amigo.geneontology.org/amigo/term/GO:1901031) | 2 out of 95 genes, 2.1% | 3 out of 490 genes, 0.6% | 0.098 |
| [negative regulation of reactive oxygen species metabolic process](http://amigo.geneontology.org/amigo/term/GO:2000378) | 2 out of 95 genes, 2.1% | 3 out of 490 genes, 0.6% | 0.098 |

GO, gene ontology; SLE, systemic lupus erythematosus.
