# Supplementary material for: Coagulation cascade and complement system in systemic lupus erythematosus
Source: Oncotarget. 2017 Dec 11;9(19):14862–81. doi: 10.18632/oncotarget.23206 (PMC5871083; doi:10.18632/oncotarget.23206)
Supplement: Supplementary file 6 [file oncotarget-09-14862-s006.docx]

**Supplementary Table 6. Selected KEGG pathway enrichments identified among** **differentially expressed proteins in SLE patients.**

| **Pathway** | **Differential proteins with pathway annotation (97)** | **All proteins with pathway annotation (539)** | ***p*-value** | **Pathway ID** |
| --- | --- | --- | --- | --- |
| [Complement and coagulation cascades](file:///C:\Users\Administrator\Desktop\文章图和表\蛋白\GO%20kegg.xlsx#RANGE!F4) | 21 (21.65%) | 78 (14.47%) | 0.023 | ko04610 |
| [Galactose metabolism](file:///C:\Users\Administrator\Desktop\文章图和表\蛋白\GO%20kegg.xlsx#RANGE!F5) | 2 (2.06%) | 3 (0.56%) | 0.085 | ko00052 |
| [Transcriptional misregulation in cancer](file:///C:\Users\Administrator\Desktop\文章图和表\蛋白\GO%20kegg.xlsx#RANGE!F6) | 11 (11.34%) | 45 (8.35%) | 0.164 | ko05202 |
| [Glycosphingolipid biosynthesis - lacto and neolacto series](file:///C:\Users\Administrator\Desktop\文章图和表\蛋白\GO%20kegg.xlsx#RANGE!F7) | 1 (1.03%) | 1 (0.19%) | 0.180 | ko00601 |
| [Glycosaminoglycan biosynthesis - keratan sulfate](file:///C:\Users\Administrator\Desktop\文章图和表\蛋白\GO%20kegg.xlsx#RANGE!F8) | 1 (1.03%) | 1 (0.19%) | 0.180 | ko00533 |
| [Other types of O-glycan biosynthesis](file:///C:\Users\Administrator\Desktop\文章图和表\蛋白\GO%20kegg.xlsx#RANGE!F9) | 1 (1.03%) | 1 (0.19%) | 0.180 | ko00514 |
| [Phenylalanine metabolism](file:///C:\Users\Administrator\Desktop\文章图和表\蛋白\GO%20kegg.xlsx#RANGE!F10) | 1 (1.03%) | 1 (0.19%) | 0.180 | ko00360 |
| [Pertussis](file:///C:\Users\Administrator\Desktop\文章图和表\蛋白\GO%20kegg.xlsx#RANGE!F11) | 6 (6.19%) | 23 (4.27%) | 0.218 | ko05133 |
| [Toll-like receptor signaling pathway](file:///C:\Users\Administrator\Desktop\文章图和表\蛋白\GO%20kegg.xlsx#RANGE!F12) | 2 (2.06%) | 5 (0.93%) | 0.222 | ko04620 |
| [Protein digestion and absorption](file:///C:\Users\Administrator\Desktop\文章图和表\蛋白\GO%20kegg.xlsx#RANGE!F13) | 2 (2.06%) | 5 (0.93%) | 0.222 | ko04974 |
| [Fat digestion and absorption](file:///C:\Users\Administrator\Desktop\文章图和表\蛋白\GO%20kegg.xlsx#RANGE!F14) | 2 (2.06%) | 6 (1.11%) | 0.295 | ko04975 |
| [Osteoclast differentiation](file:///C:\Users\Administrator\Desktop\文章图和表\蛋白\GO%20kegg.xlsx#RANGE!F15) | 1 (1.03%) | 2 (0.37%) | 0.328 | ko04380 |
| [Progesterone-mediated oocyte maturation](file:///C:\Users\Administrator\Desktop\文章图和表\蛋白\GO%20kegg.xlsx#RANGE!F16) | 1 (1.03%) | 2 (0.37%) | 0.328 | ko04914 |
| [Chemokine signaling pathway](file:///C:\Users\Administrator\Desktop\文章图和表\蛋白\GO%20kegg.xlsx#RANGE!F17) | 2 (2.06%) | 7 (1.3%) | 0.368 | ko04062 |
| [p53 signaling pathway](file:///C:\Users\Administrator\Desktop\文章图和表\蛋白\GO%20kegg.xlsx#RANGE!F18) | 3 (3.09%) | 12 (2.23%) | 0.370 | ko04115 |
| [Antigen processing and presentation](file:///C:\Users\Administrator\Desktop\文章图和表\蛋白\GO%20kegg.xlsx#RANGE!F19) | 3 (3.09%) | 12 (2.23%) | 0.370 | ko04612 |
| [TGF-beta signaling pathway](file:///C:\Users\Administrator\Desktop\文章图和表\蛋白\GO%20kegg.xlsx#RANGE!F20) | 3 (3.09%) | 13 (2.41%) | 0.424 | ko04350 |
| [Inositol phosphate metabolism](file:///C:\Users\Administrator\Desktop\文章图和表\蛋白\GO%20kegg.xlsx#RANGE!F21) | 1 (1.03%) | 3 (0.56%) | 0.449 | ko00562 |
| [Aminoacyl-tRNA biosynthesis](file:///C:\Users\Administrator\Desktop\文章图和表\蛋白\GO%20kegg.xlsx#RANGE!F22) | 1 (1.03%) | 3 (0.56%) | 0.449 | ko00970 |
| [NF-kappa B signaling pathway](file:///C:\Users\Administrator\Desktop\文章图和表\蛋白\GO%20kegg.xlsx#RANGE!F23) | 8 (8.25%) | 41 (7.61%) | 0.464 | ko04064 |
| [Rheumatoid arthritis](file:///C:\Users\Administrator\Desktop\文章图和表\蛋白\GO%20kegg.xlsx#RANGE!F24) | 8 (8.25%) | 41 (7.61%) | 0.464 | ko05323 |
| [MAPK signaling pathway](file:///C:\Users\Administrator\Desktop\文章图和表\蛋白\GO%20kegg.xlsx#RANGE!F25) | 3 (3.09%) | 14 (2.6%) | 0.476 | ko04010 |
| [Staphylococcus aureus infection](file:///C:\Users\Administrator\Desktop\文章图和表\蛋白\GO%20kegg.xlsx#RANGE!F26) | 13 (13.4%) | 70 (12.99%) | 0.502 | ko05150 |
| [Chagas disease (American trypanosomiasis)](file:///C:\Users\Administrator\Desktop\文章图和表\蛋白\GO%20kegg.xlsx#RANGE!F27) | 2 (2.06%) | 9 (1.67%) | 0.502 | ko05142 |
| [Salmonella infection](file:///C:\Users\Administrator\Desktop\文章图和表\蛋白\GO%20kegg.xlsx#RANGE!F28) | 2 (2.06%) | 9 (1.67%) | 0.502 | ko05132 |
| [Primary immunodeficiency](file:///C:\Users\Administrator\Desktop\文章图和表\蛋白\GO%20kegg.xlsx#RANGE!F29) | 16 (16.49%) | 87 (16.14%) | 0.510 | ko05340 |
| [B cell receptor signaling pathway](file:///C:\Users\Administrator\Desktop\文章图和表\蛋白\GO%20kegg.xlsx#RANGE!F30) | 7 (7.22%) | 37 (6.86%) | 0.511 | ko04662 |
| [Tuberculosis](file:///C:\Users\Administrator\Desktop\文章图和表\蛋白\GO%20kegg.xlsx#RANGE!F31) | 8 (8.25%) | 43 (7.98%) | 0.524 | ko05152 |
| [Hematopoietic cell lineage](file:///C:\Users\Administrator\Desktop\文章图和表\蛋白\GO%20kegg.xlsx#RANGE!F32) | 8 (8.25%) | 43 (7.98%) | 0.524 | ko04640 |
| [Prostate cancer](file:///C:\Users\Administrator\Desktop\文章图和表\蛋白\GO%20kegg.xlsx#RANGE!F33) | 1 (1.03%) | 4 (0.74%) | 0.549 | ko05215 |

SLE, systemic lupus erythematosus.
