# Supplementary material for: Coagulation cascade and complement system in systemic lupus erythematosus
Source: Oncotarget. 2017 Dec 11;9(19):14862–81. doi: 10.18632/oncotarget.23206 (PMC5871083; doi:10.18632/oncotarget.23206)
Supplement: Supplementary file 7 [file oncotarget-09-14862-s007.docx]

**Supplementary Table 8. KEGG pathway enrichments identified among differentially expressed metabolites in SLE patients.**

| **Pathway** | **Differential moleculars (count)** | **Pathway ID** |
| --- | --- | --- |
| [Purine metabolism](file:///C:\Users\Administrator\Desktop\文章图和表\代谢\KEGG.xlsx#RANGE!D3) | 1 | ko00230 |
| [Fluorobenzoate degradation](file:///C:\Users\Administrator\Desktop\文章图和表\代谢\KEGG.xlsx#RANGE!D4) | 2 | ko00364 |
| [Pentose phosphate pathway](file:///C:\Users\Administrator\Desktop\文章图和表\代谢\KEGG.xlsx#RANGE!D5) | 2 | ko00030 |
| [Zeatin biosynthesis](file:///C:\Users\Administrator\Desktop\文章图和表\代谢\KEGG.xlsx#RANGE!D6) | 1 | ko00908 |
| [Fatty acid biosynthesis](file:///C:\Users\Administrator\Desktop\文章图和表\代谢\KEGG.xlsx#RANGE!D7) | 1 | ko00061 |
| [Tuberculosis](file:///C:\Users\Administrator\Desktop\文章图和表\代谢\KEGG.xlsx#RANGE!D8) | 1 | ko05152 |
| [Insect hormone biosynthesis](file:///C:\Users\Administrator\Desktop\文章图和表\代谢\KEGG.xlsx#RANGE!D9) | 4 | ko00981 |
| [Atrazine degradation](file:///C:\Users\Administrator\Desktop\文章图和表\代谢\KEGG.xlsx#RANGE!D10) | 4 | ko00791 |
| [Amino sugar and nucleotide sugar metabolism](file:///C:\Users\Administrator\Desktop\文章图和表\代谢\KEGG.xlsx#RANGE!D11) | 5 | ko00520 |
| [Plant hormone signal transduction](file:///C:\Users\Administrator\Desktop\文章图和表\代谢\KEGG.xlsx#RANGE!D12) | 2 | ko04075 |
| [Starch and sucrose metabolism](file:///C:\Users\Administrator\Desktop\文章图和表\代谢\KEGG.xlsx#RANGE!D13) | 5 | ko00500 |
| [Prostate cancer](file:///C:\Users\Administrator\Desktop\文章图和表\代谢\KEGG.xlsx#RANGE!D14) | 1 | ko05215 |
| [ABC transporters](file:///C:\Users\Administrator\Desktop\文章图和表\代谢\KEGG.xlsx#RANGE!D15) | 6 | ko02010 |
| [Polycyclic aromatic hydrocarbon degradation](file:///C:\Users\Administrator\Desktop\文章图和表\代谢\KEGG.xlsx#RANGE!D16) | 7 | ko00624 |
| [Insulin signaling pathway](file:///C:\Users\Administrator\Desktop\文章图和表\代谢\KEGG.xlsx#RANGE!D17) | 1 | ko04910 |
| [Linoleic acid metabolism](file:///C:\Users\Administrator\Desktop\文章图和表\代谢\KEGG.xlsx#RANGE!D18) | 2 | ko00591 |
| [Biosynthesis of 12-, 14- and 16-membered macrolides](file:///C:\Users\Administrator\Desktop\文章图和表\代谢\KEGG.xlsx#RANGE!D19) | 11 | ko00522 |
| [Lysine degradation](file:///C:\Users\Administrator\Desktop\文章图和表\代谢\KEGG.xlsx#RANGE!D20) | 2 | ko00310 |
| [Mineral absorption](file:///C:\Users\Administrator\Desktop\文章图和表\代谢\KEGG.xlsx#RANGE!D21) | 6 | ko04978 |
| [Porphyrin and chlorophyll metabolism](file:///C:\Users\Administrator\Desktop\文章图和表\代谢\KEGG.xlsx#RANGE!D22) | 1 | ko00860 |
| [Bacterial chemotaxis](file:///C:\Users\Administrator\Desktop\文章图和表\代谢\KEGG.xlsx#RANGE!D23) | 1 | ko02030 |
| [Limonene and pinene degradation](file:///C:\Users\Administrator\Desktop\文章图和表\代谢\KEGG.xlsx#RANGE!D24) | 8 | ko00903 |
| [Tropane, piperidine and pyridine alkaloid biosynthesis](file:///C:\Users\Administrator\Desktop\文章图和表\代谢\KEGG.xlsx#RANGE!D25) | 2 | ko00960 |
| [Bile secretion](file:///C:\Users\Administrator\Desktop\文章图和表\代谢\KEGG.xlsx#RANGE!D26) | 4 | ko04976 |
| [Inositol phosphate metabolism](file:///C:\Users\Administrator\Desktop\文章图和表\代谢\KEGG.xlsx#RANGE!D27) | 3 | ko00562 |
| [Phosphotransferase system (PTS)](file:///C:\Users\Administrator\Desktop\文章图和表\代谢\KEGG.xlsx#RANGE!D28) | 4 | ko02060 |
| [Betalain biosynthesis](file:///C:\Users\Administrator\Desktop\文章图和表\代谢\KEGG.xlsx#RANGE!D29) | 1 | ko00965 |
| [Pantothenate and CoA biosynthesis](file:///C:\Users\Administrator\Desktop\文章图和表\代谢\KEGG.xlsx#RANGE!D30) | 2 | ko00770 |
| [Methane metabolism](file:///C:\Users\Administrator\Desktop\文章图和表\代谢\KEGG.xlsx#RANGE!D31) | 1 | ko00680 |
| [Carbohydrate digestion and absorption](file:///C:\Users\Administrator\Desktop\文章图和表\代谢\KEGG.xlsx#RANGE!D32) | 3 | ko04973 |
| [Carotenoid biosynthesis](file:///C:\Users\Administrator\Desktop\文章图和表\代谢\KEGG.xlsx#RANGE!D33) | 4 | ko00906 |
| [African trypanosomiasis](file:///C:\Users\Administrator\Desktop\文章图和表\代谢\KEGG.xlsx#RANGE!D34) | 2 | ko05143 |
| [Nitrotoluene degradation](file:///C:\Users\Administrator\Desktop\文章图和表\代谢\KEGG.xlsx#RANGE!D35) | 1 | ko00633 |
| [Biosynthesis of secondary metabolites](file:///C:\Users\Administrator\Desktop\文章图和表\代谢\KEGG.xlsx#RANGE!D36) | 56 | ko01110 |
| [Steroid hormone biosynthesis](file:///C:\Users\Administrator\Desktop\文章图和表\代谢\KEGG.xlsx#RANGE!D37) | 12 | ko00140 |
| [Phosphonate and phosphinate metabolism](file:///C:\Users\Administrator\Desktop\文章图和表\代谢\KEGG.xlsx#RANGE!D38) | 2 | ko00440 |
| [Glycolysis / Gluconeogenesis](file:///C:\Users\Administrator\Desktop\文章图和表\代谢\KEGG.xlsx#RANGE!D39) | 3 | ko00010 |
| [Ether lipid metabolism](file:///C:\Users\Administrator\Desktop\文章图和表\代谢\KEGG.xlsx#RANGE!D40) | 1 | ko00565 |
| [Primary bile acid biosynthesis](file:///C:\Users\Administrator\Desktop\文章图和表\代谢\KEGG.xlsx#RANGE!D41) | 5 | ko00120 |
| [Microbial metabolism in diverse environments](file:///C:\Users\Administrator\Desktop\文章图和表\代谢\KEGG.xlsx#RANGE!D42) | 42 | ko01120 |
| [Endocrine and other factor-regulated calcium reabsorption](file:///C:\Users\Administrator\Desktop\文章图和表\代谢\KEGG.xlsx#RANGE!D43) | 1 | ko04961 |
| [Histidine metabolism](file:///C:\Users\Administrator\Desktop\文章图和表\代谢\KEGG.xlsx#RANGE!D44) | 3 | ko00340 |
| [Metabolic pathways](file:///C:\Users\Administrator\Desktop\文章图和表\代谢\KEGG.xlsx#RANGE!D45) | 68 | ko01100 |
| [Biosynthesis of unsaturated fatty acids](file:///C:\Users\Administrator\Desktop\文章图和表\代谢\KEGG.xlsx#RANGE!D46) | 1 | ko01040 |
| [Monoterpenoid biosynthesis](file:///C:\Users\Administrator\Desktop\文章图和表\代谢\KEGG.xlsx#RANGE!D47) | 5 | ko00902 |
| [Glucosinolate biosynthesis](file:///C:\Users\Administrator\Desktop\文章图和表\代谢\KEGG.xlsx#RANGE!D48) | 7 | ko00966 |
| [Phenylalanine, tyrosine and tryptophan biosynthesis](file:///C:\Users\Administrator\Desktop\文章图和表\代谢\KEGG.xlsx#RANGE!D49) | 4 | ko00400 |
| [Butanoate metabolism](file:///C:\Users\Administrator\Desktop\文章图和表\代谢\KEGG.xlsx#RANGE!D50) | 1 | ko00650 |
| [Ascorbate and aldarate metabolism](file:///C:\Users\Administrator\Desktop\文章图和表\代谢\KEGG.xlsx#RANGE!D51) | 3 | ko00053 |
| [Caprolactam degradation](file:///C:\Users\Administrator\Desktop\文章图和表\代谢\KEGG.xlsx#RANGE!D52) | 7 | ko00930 |
| [Flavone and flavonol biosynthesis](file:///C:\Users\Administrator\Desktop\文章图和表\代谢\KEGG.xlsx#RANGE!D53) | 2 | ko00944 |
| [Benzoxazinoid biosynthesis](file:///C:\Users\Administrator\Desktop\文章图和表\代谢\KEGG.xlsx#RANGE!D54) | 1 | ko00402 |
| [Geraniol degradation](file:///C:\Users\Administrator\Desktop\文章图和表\代谢\KEGG.xlsx#RANGE!D55) | 1 | ko00281 |
| [Aminoacyl-tRNA biosynthesis](file:///C:\Users\Administrator\Desktop\文章图和表\代谢\KEGG.xlsx#RANGE!D56) | 3 | ko00970 |
| [Metabolism of xenobiotics by cytochrome P450](file:///C:\Users\Administrator\Desktop\文章图和表\代谢\KEGG.xlsx#RANGE!D57) | 2 | ko00980 |
| [Progesterone-mediated oocyte maturation](file:///C:\Users\Administrator\Desktop\文章图和表\代谢\KEGG.xlsx#RANGE!D58) | 1 | ko04914 |
| [Protein digestion and absorption](file:///C:\Users\Administrator\Desktop\文章图和表\代谢\KEGG.xlsx#RANGE!D59) | 5 | ko04974 |
| [Aminobenzoate degradation](file:///C:\Users\Administrator\Desktop\文章图和表\代谢\KEGG.xlsx#RANGE!D60) | 2 | ko00627 |
| [Xylene degradation](file:///C:\Users\Administrator\Desktop\文章图和表\代谢\KEGG.xlsx#RANGE!D61) | 5 | ko00622 |
| [Sphingolipid metabolism](file:///C:\Users\Administrator\Desktop\文章图和表\代谢\KEGG.xlsx#RANGE!D62) | 1 | ko00600 |
| [Type II diabetes mellitus](file:///C:\Users\Administrator\Desktop\文章图和表\代谢\KEGG.xlsx#RANGE!D63) | 1 | ko04930 |
| [Brassinosteroid biosynthesis](file:///C:\Users\Administrator\Desktop\文章图和表\代谢\KEGG.xlsx#RANGE!D64) | 9 | ko00905 |
| [Indole alkaloid biosynthesis](file:///C:\Users\Administrator\Desktop\文章图和表\代谢\KEGG.xlsx#RANGE!D65) | 5 | ko00901 |
| [Vitamin digestion and absorption](file:///C:\Users\Administrator\Desktop\文章图和表\代谢\KEGG.xlsx#RANGE!D66) | 2 | ko04977 |
| [Styrene degradation](file:///C:\Users\Administrator\Desktop\文章图和表\代谢\KEGG.xlsx#RANGE!D67) | 2 | ko00643 |
| [Isoquinoline alkaloid biosynthesis](file:///C:\Users\Administrator\Desktop\文章图和表\代谢\KEGG.xlsx#RANGE!D68) | 2 | ko00950 |
| [Neuroactive ligand-receptor interaction](file:///C:\Users\Administrator\Desktop\文章图和表\代谢\KEGG.xlsx#RANGE!D69) | 1 | ko04080 |
| [Arginine and proline metabolism](file:///C:\Users\Administrator\Desktop\文章图和表\代谢\KEGG.xlsx#RANGE!D70) | 3 | ko00330 |
| [Tryptophan metabolism](file:///C:\Users\Administrator\Desktop\文章图和表\代谢\KEGG.xlsx#RANGE!D71) | 9 | ko00380 |
| [Stilbenoid, diarylheptanoid and gingerol biosynthesis](file:///C:\Users\Administrator\Desktop\文章图和表\代谢\KEGG.xlsx#RANGE!D72) | 1 | ko00945 |
| [Meiosis - yeast](file:///C:\Users\Administrator\Desktop\文章图和表\代谢\KEGG.xlsx#RANGE!D73) | 1 | ko04113 |
| [Biosynthesis of ansamycins](file:///C:\Users\Administrator\Desktop\文章图和表\代谢\KEGG.xlsx#RANGE!D74) | 1 | ko01051 |
| [Phenylalanine metabolism](file:///C:\Users\Administrator\Desktop\文章图和表\代谢\KEGG.xlsx#RANGE!D75) | 4 | ko00360 |
| [Galactose metabolism](file:///C:\Users\Administrator\Desktop\文章图和表\代谢\KEGG.xlsx#RANGE!D76) | 7 | ko00052 |
| [Phosphatidylinositol signaling system](file:///C:\Users\Administrator\Desktop\文章图和表\代谢\KEGG.xlsx#RANGE!D77) | 1 | ko04070 |
| [Chlorocyclohexane and chlorobenzene degradation](file:///C:\Users\Administrator\Desktop\文章图和表\代谢\KEGG.xlsx#RANGE!D78) | 1 | ko00361 |
| [Steroid biosynthesis](file:///C:\Users\Administrator\Desktop\文章图和表\代谢\KEGG.xlsx#RANGE!D79) | 3 | ko00100 |
| [Lysine biosynthesis](file:///C:\Users\Administrator\Desktop\文章图和表\代谢\KEGG.xlsx#RANGE!D80) | 1 | ko00300 |
| [Pathways in cancer](file:///C:\Users\Administrator\Desktop\文章图和表\代谢\KEGG.xlsx#RANGE!D81) | 1 | ko05200 |
| [Glycerophospholipid metabolism](file:///C:\Users\Administrator\Desktop\文章图和表\代谢\KEGG.xlsx#RANGE!D82) | 3 | ko00564 |
| [Oocyte meiosis](file:///C:\Users\Administrator\Desktop\文章图和表\代谢\KEGG.xlsx#RANGE!D83) | 1 | ko04114 |
| [Complement and coagulation cascades](file:///C:\Users\Administrator\Desktop\文章图和表\代谢\KEGG.xlsx#RANGE!D84) | 1 | ko04610 |
| [Valine, leucine and isoleucine degradation](file:///C:\Users\Administrator\Desktop\文章图和表\代谢\KEGG.xlsx#RANGE!D85) | 3 | ko00280 |
| [Fructose and mannose metabolism](file:///C:\Users\Administrator\Desktop\文章图和表\代谢\KEGG.xlsx#RANGE!D86) | 6 | ko00051 |
| [Biotin metabolism](file:///C:\Users\Administrator\Desktop\文章图和表\代谢\KEGG.xlsx#RANGE!D87) | 1 | ko00780 |
| [Proximal tubule bicarbonate reclamation](file:///C:\Users\Administrator\Desktop\文章图和表\代谢\KEGG.xlsx#RANGE!D88) | 1 | ko04964 |
| [Cyanoamino acid metabolism](file:///C:\Users\Administrator\Desktop\文章图和表\代谢\KEGG.xlsx#RANGE!D89) | 1 | ko00460 |
| [Naphthalene degradation](file:///C:\Users\Administrator\Desktop\文章图和表\代谢\KEGG.xlsx#RANGE!D90) | 2 | ko00626 |
| [Lysosome](file:///C:\Users\Administrator\Desktop\文章图和表\代谢\KEGG.xlsx#RANGE!D91) | 1 | ko04142 |
| [Valine, leucine and isoleucine biosynthesis](file:///C:\Users\Administrator\Desktop\文章图和表\代谢\KEGG.xlsx#RANGE!D92) | 4 | ko00290 |
| [beta-Alanine metabolism](file:///C:\Users\Administrator\Desktop\文章图和表\代谢\KEGG.xlsx#RANGE!D93) | 1 | ko00410 |
| [Retinol metabolism](file:///C:\Users\Administrator\Desktop\文章图和表\代谢\KEGG.xlsx#RANGE!D94) | 2 | ko00830 |
| [Ethylbenzene degradation](file:///C:\Users\Administrator\Desktop\文章图和表\代谢\KEGG.xlsx#RANGE!D95) | 2 | ko00642 |
| [Two-component system](file:///C:\Users\Administrator\Desktop\文章图和表\代谢\KEGG.xlsx#RANGE!D96) | 2 | ko02020 |
| [Streptomycin biosynthesis](file:///C:\Users\Administrator\Desktop\文章图和表\代谢\KEGG.xlsx#RANGE!D97) | 2 | ko00521 |
| [Regulation of autophagy](file:///C:\Users\Administrator\Desktop\文章图和表\代谢\KEGG.xlsx#RANGE!D98) | 1 | ko04140 |
| [Caffeine metabolism](file:///C:\Users\Administrator\Desktop\文章图和表\代谢\KEGG.xlsx#RANGE!D99) | 5 | ko00232 |
| [Butirosin and neomycin biosynthesis](file:///C:\Users\Administrator\Desktop\文章图和表\代谢\KEGG.xlsx#RANGE!D100) | 3 | ko00524 |
| [Glycine, serine and threonine metabolism](file:///C:\Users\Administrator\Desktop\文章图和表\代谢\KEGG.xlsx#RANGE!D101) | 1 | ko00260 |
| [Tyrosine metabolism](file:///C:\Users\Administrator\Desktop\文章图和表\代谢\KEGG.xlsx#RANGE!D102) | 4 | ko00350 |
| [Drug metabolism - cytochrome P450](file:///C:\Users\Administrator\Desktop\文章图和表\代谢\KEGG.xlsx#RANGE!D103) | 9 | ko00982 |

SLE, systemic lupus erythematosus.
